# Alpha and Beta Emitters in Translational Nuclear Medicine: Clinical Advances, Challenges, and Future Direction

**DOI:** 10.3390/ijms27052290

**Published:** 2026-02-28

**Authors:** Hanieh Karimi, Thomas H. Shaffer, Erik Stauff, Vinay V. R. Kandula, Heidi H. Kecskemethy, Lauren W. Averill, Xuyi Yue

**Affiliations:** 1 Department of Radiology, Nemours Children’s Health, Wilmington, DE 19803, USA; hanieh.karimi@nemours.org (H.K.); erik.stauff@nemours.org (E.S.); vinay.kandula@nemours.org (V.V.R.K.); heidi.kecskemethy@nemours.org (H.H.K.); lauren.averill@nemours.org (L.W.A.); 2 Diagnostic & Research PET/MR Center, Nemours Children’s Health, Wilmington, DE 19803, USA; 3 Nemours Biomedical Research, Nemours Children’s Health, Wilmington, DE 19803, USA; thomas.shaffer@nemours.org; 4 Department of Radiology, Thomas Jefferson University, Philadelphia, PA 19107, USA; 5 Department of Psychological and Brain Sciences, University of Delaware, Newark, DE 19716, USA

**Keywords:** radiopharmaceutical therapy (RPT), alpha emitters, beta emitters, linear energy transfer (LET), radiotheranostics, molecular targeting, clinical investigation

## Abstract

Radiopharmaceutical therapy (RPT) has emerged as a transformative modality in oncology, particularly for patients with metastatic or inoperable tumors. By leveraging molecularly targeted carriers conjugated to cytotoxic radionuclides, RPT enables precise delivery of ionizing radiation to tumor sites while minimizing off-target effects. Central to this approach are alpha (α) and beta (β) particle-emitting radionuclides. This review aims to provide a comprehensive overview of all clinically relevant alpha and beta emitters and incorporates the most recent advances from 2017–2025, offering a comprehensive and up-to-date perspective. Alpha and beta emitters hold significant promises for the future, especially in nuclear medicine, energy, and environmental monitoring. Medically, these emitters are at the forefront of targeted radiotherapy, offering new hope for cancer treatment. Alpha emitters such as Actinium-225 and Radium-223 are gaining attention for their high linear energy transfer, which allows them to effectively kill cancer cells while minimizing damage to surrounding healthy tissues. Beta emitters, including Lutetium-177 and Iodine-131, are already widely used for treating thyroid cancer, neuroendocrine tumors, and prostate cancer. They offer a longer range in tissue penetration than alpha particles, making them suitable for larger or more diffuse tumors. Alpha and beta emitters hold tremendous promise in targeted radiotherapy. However, current research is limited by an incomplete understanding of resistance pathways, insufficient long-term safety and efficacy data, and underdeveloped personalized treatment frameworks. As production technologies improve and safety protocols advance, these emitters will likely play an even more prominent role in both health care and scientific innovation.

## 1. Introduction

Radiopharmaceutical therapy, which harnesses the cytotoxic potential of radionuclide-labeled compounds, has become an increasingly pivotal approach in the management of cancer, particularly for patients with disseminated or non-operable tumors. Central to this strategy are alpha (α) and beta (β) particle-emitting radionuclides, each offering distinct physical properties, biological effects, and therapeutic advantages [1]. Unlike conventional chemotherapy or external beam radiation, targeted radionuclide therapy (TRT) makes use of molecular targeting vectors such as antibodies, peptides, or small molecules to selectively bind tumor-associated antigens or receptors and deliver therapeutic payloads of radioactivity [2]. The physical and biological differences between alpha and beta emitters offer complementary therapeutic profiles, allowing treatment customization based on tumor size, burden, and radiosensitivity [1,2].

Alpha emitters such as ^223^Ra, ^225^Ac, and ^211^At deliver high linear energy transfer ([LET]: ~50–230 keV/μm) radiation over a short range (28–100 µm), causing dense ionization that induces double-stranded DNA breaks with high cytotoxicity and minimal collateral damage [3]. The clinical value of alpha therapy was firmly established by the pivotal phase III ALpharadin in SYMPtomatic Prostate Cancer (ALSYMPCA) trial, which evaluated ^223^Ra-dichloride in patients with metastatic castration-resistant prostate cancer (mCRPC) and symptomatic bone metastases. In this randomized, double-blind, placebo-controlled study, radium-223 significantly improved overall survival (OS) (14.9 vs. 11.3 months; HR 0.70) and delayed time to first symptomatic skeletal event. It also demonstrated a favorable safety profile. Importantly, ALSYMPCA was the first trial to show an overall survival benefit with a bone-targeted radiopharmaceutical, leading to regulatory approval and incorporation into international treatment guidelines. This study marked a paradigm shift by validating targeted alpha therapy as a life-prolonging systemic treatment rather than merely a palliative option [4]. Building on these advances, targeted alpha therapy has since advanced with ^225^Ac-labeled prostate-specific membrane antigen (PSMA) ligands. While these developments are supported primarily by phase I/II and retrospective studies, [^225^Ac]Ac-PSMA-617 has demonstrated remarkable biochemical and radiographic response rates in patients progressing after beta-emitting PSMA therapy. These findings build upon the treatment landscape shaped by the phase III VISION trial, which established [^177^Lu]Lu-PSMA-617 as a standard-of-care therapy in PSMA-positive mCRPC. In VISION, the addition of [^177^Lu]Lu-PSMA-617 to standard care significantly improved both radiographic progression-free survival (8.7 vs. 3.4 months) and overall survival (15.3 vs. 11.3 months; HR 0.62), leading to regulatory approval and widespread clinical adoption. The success of VISION not only validated PSMA as a therapeutic target but also paved the way for the subsequent development of alpha-emitting PSMA-directed therapies [5]. Additionally, ^211^At-labeled agents are being clinically studied in glioblastoma, ovarian cancer, and leukemia due to their potent anti-tumor activity and favorable pharmacokinetics [6].

Beta-emitting radionuclides such as ^131^I, ^90^Y, and ^177^Lu have been used clinically for decades. The relatively long path length of beta particles (ranging from 2 mm to 12 mm) and moderate LET (~0.2 keV/μm) enable them to treat large tumor masses through a crossfire effect [1,3]. A landmark in beta-emitter therapy was the phase III Neuroendocrine Tumors Therapy (NETTER-1) trial. This study evaluated [^177^Lu]Lu-DOTATATE in patients with advanced midgut neuroendocrine tumors. The randomized trial showed a 79% reduction in the risk of disease progression or death compared with high-dose octreotide. It also achieved a significantly improved objective response rate and manageable toxicity. NETTER-1 established peptide receptor radionuclide therapy (PRRT) as a new standard of care in somatostatin receptor–positive neuroendocrine tumors and led to global regulatory approval. The trial provided high-level evidence that molecularly targeted radioligand therapy could offer durable disease control with acceptable safety in solid tumors [7].

Together, ALSYMPCA, NETTER-1, and VISION represent transformative milestones in radiopharmaceutical therapy. These pivotal randomized phase III trials provided definitive survival data, secured regulatory approvals, and firmly integrated radioligand therapy into contemporary oncologic treatment algorithms [8]. While early-phase and exploratory studies continue to refine dosing strategies, explore combination regimens, and expand indications, these landmark trials established the evidence-based foundation for the current field of radiotheranostics [9].

Despite these advances, several challenges persist, including supply limitations of alpha emitters (e.g., ^225^Ac), toxicity management (e.g., xerostomia with salivary PSMA expression), and the need for individualized dosimetry [10]. Current clinical trials are increasingly focused on optimizing therapeutic indices, exploring novel combinations (e.g., with immunotherapy or poly (ADP-ribose) polymerase (PARP) inhibitors), and expanding indications to solid tumors and hematological malignancies [11,12,13]. Ongoing trials such as SPLASH, ECLIPSE, and ACCURET are further validating the clinical value of these agents in broader patient populations [14,15].

This review provides a comprehensive overview of all clinically relevant alpha and beta emitters and incorporates the most recent advances from 2017–2025 and offers a comprehensive and up-to-date perspective. Unlike previous reports that focus on limited numbers of radionuclides or short time frames, this report compares both particle types, highlights emerging clinical data, and explores future directions in radiopharmaceutical therapy and precision oncology.

### 1.1. Physical and Biological Properties

**Figure 1:**
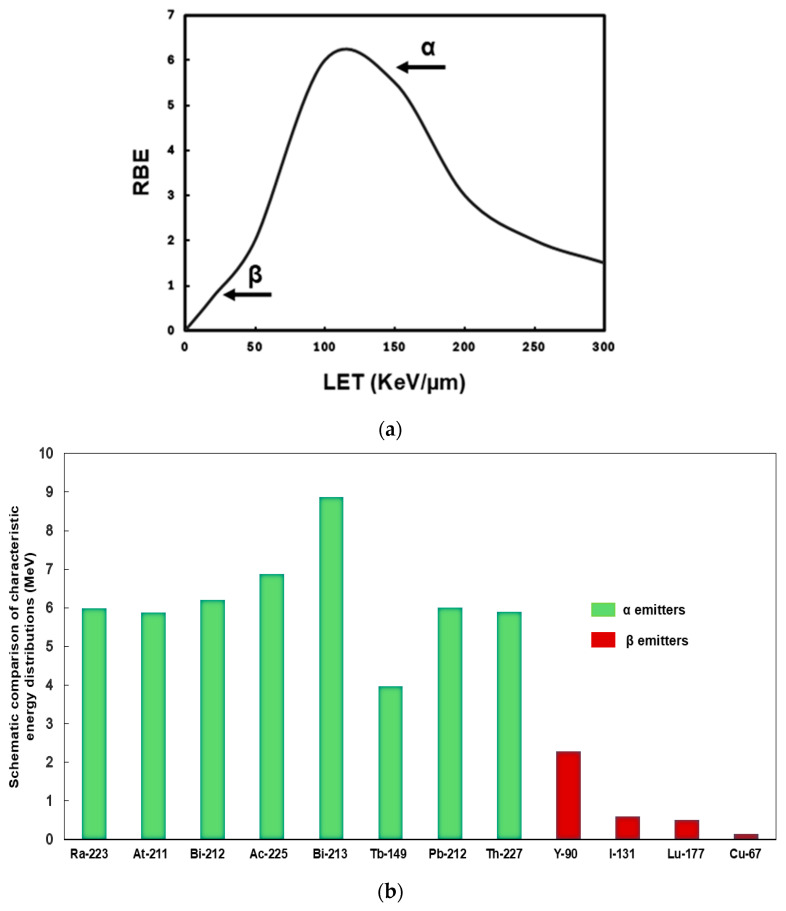
(**a**) Alpha vs. beta emitters: alpha emitters (Radium-223, Astatine-211, Bismuth-212, Actinium-225, Bismuth-213, Terbium-149, Lead-212, Thorium-227) with their high LET, deposit concentrated energy leading to a higher relative biological effectiveness when compared with beta emitters (Yttrium-90, Iodine-131, Lutetium-177, Copper-67) which have a lower linear energy transfer and therefore a less localized energy deposition pattern. (**b**) Schematic comparison of characteristic energy distributions of alpha and beta particles emitted during radioactive decay.

Alpha emitters release heavy, positively charged particles (helium nuclei) with high LET (~50–230 keV/µm), resulting in densely ionizing tracks that induce irreparable double-stranded DNA breaks. Their path length in tissue is extremely short (28–100 µm), limiting damage to surrounding healthy cells and making them ideal for treating minimal residual diseases, micrometastases, and single cancer cells [3]. They have high relative biological effectiveness (RBE) and short-range reduce bystander effects, but they require highly specific targeting due to lack of crossfire [16] (Figure 1).

Beta emitters, in contrast, are lightweight, negatively charged electrons with lower LET (~0.2 keV/µm) and longer tissue penetration (2–12 mm) (Figure 1). This enables the treatment of larger or heterogeneous tumors via the crossfire effect, compensating for heterogeneous target expression. However, the damage is often limited to single-strand DNA breaks, leading to lower cytotoxic efficiency per decay [1,3] (Table 1) [17,18,19,20,21,22,23,24,25,26,27,28,29,30,31,32,33,34,35,36,37,38,39,40,41,42,43,44,45,46]. In clinical applications, alpha emitters are favored for precision in small-volume lesions, while beta emitters remain a mainstay for larger tumor burdens. Understanding these properties is critical when selecting the appropriate radionuclides for therapeutic use [47].

### 1.2. Radiopharmaceutical Development

In this section, radiopharmaceuticals are summarized based on the type of particle emission, alpha or beta. We discuss how these radioactive isotopes are used in clinical or preclinical studies, focusing on their properties, mechanisms of action, and applications in diagnosis and therapy. Alpha emitters are used mainly for targeted cancer treatment due to their high energy and short range, while beta emitters are applied commonly in both imaging and therapeutic procedures.

### 1.3. Alpha Emitters

#### 1.3.1. Radium-223

Abramenkovs et al. [48] conducted an in vitro investigation of the cytotoxic effects of Radium-223 dichloride (Ra-223), an alpha-emitting radiopharmaceutical approved for mCRPC with bone involvement. Using PC3, DU145, and 22Rv1 prostate cancer cell lines in both two-dimensional and three-dimensional (3D) culture models, the study showed that Ra-223 exhibits an exceptionally high affinity for hydroxyapatite (HAp), a key bone matrix component, with a dissociation constant of 19.2 × 10^−18^ M. Minimal uptake by cancer cells indicated that cytotoxicity occurs mainly through proximity-based alpha irradiation rather than internalization. Exposure to Ra-223 caused extensive DNA double-strand breaks in linear, track-like patterns characteristic of alpha particles and triggered phosphorylation of DNA-dependent protein kinase catalytic subunit (DNA-PKcs) at threonine on position 2609 (T2609), confirming activation of DNA repair pathways.

Ra-223 treatment markedly reduced cell viability and colony formation, with survival decreasing more than 100-fold when cells were cultured on Ra-223-preloaded HAp when compared with equivalent concentrations in suspension. Apoptosis rates rose dramatically, exceeding 55% in treated groups, and these effects were observed in both androgen receptor splice variant 7 (ARv7)-positive and negative cell lines, demonstrating independence from androgen receptor signaling. In 3D spheroids, Ra-223 reduced spheroid volume by 50% and induced deep DNA damage penetration. Overall, the study [46] highlights Ra-223’s potent anti-tumor activity through clustered, irreparable DNA damage and its strong, persistent binding to bone-like surfaces, reinforcing its therapeutic value in targeting skeletal metastases.

Bannik et al. [49] comprehensively evaluated the radiobiological effects of Ra-223 on cancer cells using a novel Transwell-based in vitro system (Corning Life Sciences, Glendale, AZ), which enabled spatially controlled alpha particle exposure. The study involved multiple human cancer cell lines, including NCI-H460 and NCI-H12999 (human non-small cell lung cancer), 22Rv1 (human prostate carcinoma), OVCAR-3 (human ovarian carcinoma), HCT116 (human colorectal cancer), and A549 (lung carcinoma). Exposure to Ra-223 ranged from 2 to 8 h with corresponding activity concentrations between 1.3 to 2.6 kBq/cm^2^, resulting in average absorbed doses from 4.1 ± 0.2 Gy at 2 h to 16.4 ± 0.9 Gy at 8 h, as calculated using Monte Carlo-based simulations.

DNA damage, measured by tumor protein p53 binding protein 1 (53BP1) staining, increased sharply with dose and time, rising from fewer than 4 foci per nucleus in controls to more than 20 foci at higher exposures. Ra-223 induced a strong, dose-dependent G2/M cell cycle arrest, with HCT116 cells showing the highest sensitivity and accumulating up to 68% in G2/M phase at 8 h even at lower doses, indicating activation of DNA damage checkpoints. Prolonged or higher-dose Ra-223 exposure caused significant loss of cell survival, dropping to 11% in the most sensitive cell lines (HCT116 and 22Rv1) after 8 h, while more resistant cells (H1299, A549) retained 21–30% viability. Comet assays confirmed extensive, non-repairable DNA fragmentation, with tail intensities nearly tripling at the highest dose. These findings demonstrate that Ra-223 delivers potent, time- and dose-dependent alpha radiation that drives complex DNA double-strand breaks, cell cycle arrest, and clonogenic death, supporting its use as a highly cytotoxic agent for targeted alpha therapy in bone-metastatic cancers.

Shimada et al. [50] investigated the efficacy of combining Radium-223 dichloride with ethinylestradiol (EE) (EstRadium) in patients with bone mCRPC. The study included 31 patients who had received at least three doses of Ra-223 and were divided into two cohorts: 18 patients received Ra-223 in combination with EE (EstRadium group), and 13 patients received Ra-223 monotherapy (non-EstRadium group). The primary endpoint was the reduction in prostate-specific antigen (PSA) levels, a commonly used biomarker for prostate cancer activity. Notably, 12 of 18 patients (67%) in the EstRadium group experienced a decrease in PSA levels during treatment, compared with only three of 13 patients (23%) in the non-EstRadium group—a statistically significant difference (*p* = 0.029).

Additional analysis showed that the median PSA at baseline in the EstRadium group was 41.8 ng/mL (range: 0.41–202) compared with 15.5 ng/mL (range: 0.04–437) in the non-EstRadium group, indicating a comparable starting disease burden. Both groups had similar baseline characteristics in terms of age, extent of bone metastases, and prior therapies, supporting the reliability of the comparison. Importantly, no significant increase in adverse events such as bone marrow suppression or gastrointestinal toxicity was observed in the combination group, suggesting that the addition of EE does not compromise the safety profile of Ra-223. This study did not show any significant increase in survival rate between two groups. The study concludes that EE may potentiate the anti-tumor efficacy of Ra-223 in patients with mCRPC, particularly in biochemical response, and supports further investigation of this combination in larger prospective trials.

Ueno et al. [51] conducted a single-arm phase 2 clinical trial to evaluate the efficacy and safety of combining Ra-223 with standard hormonal therapy in patients with hormone receptor-positive (HR+), bone-dominant metastatic breast cancer. A total of 36 patients were enrolled; all of whom had bone metastases without evidence of visceral-dominant disease. Median age was 58 years (range: 31–79), and 69% of the patients were postmenopausal. Ra-223 was administered intravenously at a dose of 55 kBq/kg every four weeks for up to six cycles, in conjunction with continued endocrine therapy.

The primary endpoint, disease control rate at nine months, was achieved in 49% of patients (*n* = 18/36), with a median progression-free survival (PFS) of 7.4 months (95% confidence interval [CI]). Notably, bone-specific PFS was longer, with a median of 16.0 months (95% CI), suggesting Ra-223’s targeted efficacy in bone lesions. Tumor response by response evaluation criteria in solid tumors (RECIST) criteria showed that 21% of patients (*n* = 7) achieved complete response and 32% (*n* = 11) had partial response at six months, yielding an overall response rate of 54%. Regarding safety, the combination was well-tolerated, with no grade 4 or 5 adverse events reported. All grade toxicities were limited to some adverse events such as neutropenia (25%), anemia (17%), and thrombocytopenia (11%), with no treatment-related deaths. These findings demonstrate that Ra-223 combined with hormonal therapy is not only feasible and safe but also shows promising clinical activity in patients with HR+ bone-dominant metastatic breast cancer.

Raval et al. [52] performed a real-world analysis of treatment patterns and overall survival (rwOS) in 1376 men with mCRPC who received Ra-223 in the United States between January 1, 2017, and June 30, 2022. Median age of patients was 68 years; and the majority (89%) had bone-only metastases. In terms of treatment sequencing, Ra-223 was administered as first-line therapy in 17% of cases, second-line in 35%, and third-line in 25%. Combination or layered therapy primarily with enzalutamide was used in 36% of patients, and 46% completed five or more Ra-223 treatment cycles.

Median rwOS for the overall cohort was 22.9 months. Patients who completed five or more cycles had a significantly improved median rwOS of 30.3 months when compared with 15.3 months for those who received one to four cycles. Similarly, median rwOS was higher in patients receiving combination or layered therapy (26.6 months) than those on Ra-223 monotherapy (20.5 months); the former shows 22% reduction in death. Multivariable Cox regression analysis showed a 55% reduction in mortality risk for patients completing five or more Ra-223 cycles (hazard ratio [HR] = 0.45) and a 22% reduction in those undergoing combination therapy (HR = 0.78). These findings highlight the clinical value of completing Ra-223 therapy and incorporating combination strategies to enhance survival outcomes in men with mCRPC.

Ito et al. [53] evaluated the appropriate clinical use of Ra-223 for treating mCRPC; 67 patients with symptomatic bone metastases were analyzed across two Japanese centers between 2016 and 2020. The OS from the time of mCRPC diagnosis was 3.82 years. Baseline clinical factors were assessed to identify predictors of survival after Ra-223 therapy. Among these, elevated alkaline phosphatase (ALP), bone scan index (BSI) ≥ 2, and prostate-specific antigen doubling time (PSA-DT) less than three months were associated with poorer outcomes in univariate analyses. Importantly, multivariate analysis confirmed that only elevated ALP at the start of Ra-223 treatment was an independent predictor of increased mortality. Interestingly, discontinuation of Ra-223 before completing all treatment cycles did not significantly affect OS, suggesting that pre-treatment disease characteristics may be more critical than treatment completion status.

Further subgroup analysis revealed that patients with lower disease burden—specifically, BSI < 2, PSA-DT ≥ 3 months, and normal ALP experienced significantly better survival outcomes following Ra-223 therapy. These findings underscore the importance of selecting patients with favorable baseline characteristics to maximize therapeutic benefit from Ra-223. The study supports the notion that Ra-223 should ideally be administered earlier in the treatment course, prior to the onset of high tumor burden or biochemical progression, particularly elevated ALP. Collectively, this evidence contributes to optimizing treatment sequencing in mCRPC and highlights the potential survival advantage of incorporating Ra-223 at a clinically appropriate disease stage.

Wang et al. [54] investigated the RAVENS phase 2 randomized trial regarding whether the addition of Ra-223 to stereotactic ablative radiotherapy (SABR) could improve outcomes for men with oligometastatic castration-sensitive prostate cancer involving one to five bone lesions. A total of 64 patients were enrolled between 2019 and 2023 and randomized into two arms: SABR alone (*n* = 33), and SABR combined with Ra-223 (55 kBq/kg every 4 weeks for 6 cycles, *n* = 31). The median composite PFS, which included biochemical failure, progression, death, or need for androgen deprivation therapy (ADT), was 11.8 months in the SABR-only arm and 10.5 months in the SABR + Ra-223 arm. The difference was not statistically significant (adjusted hazard ratio [aHR] 1.42; 95% CI; *p* = 0.24). Similarly, metastasis-free survival (aHR 1.09; 95% CI; *p* = 0.84) and ADT-free survival (aHR 1.53; 95% CI; *p* = 0.30) did not differ significantly between groups.

In terms of safety, Ra-223 was relatively well tolerated, although grade 3 adverse events occurred in 17% of patients in the Ra-223 group versus 6% in the SABR-alone group. These events primarily included lymphopenia and musculoskeletal pain, with no grade 4 or 5 toxicities observed. Importantly, exploratory biomarker analyses revealed that patients with pathogenic mutations in DNA damage repair genes (e.g., BRCA1/2, ATM, TP53, or RB1) had significantly worse PFS (HR 5.95; 95% CI: 1.83–19.3; *p* = 0.003). In contrast, a broader baseline T-cell receptor repertoire was associated with improved PFS (aHR 0.45; *p* = 0.04), suggesting potential immune-related prognostic value. Overall, while Ra-223 did not enhance clinical outcomes in this setting, the trial identified molecular subgroups that may benefit from future treatment personalization strategies.

Wenzel et al. [55] evaluated the impact of prior Ra-223 therapy on the efficacy of subsequent [^177^Lu]Lu-PSMA radioligand treatment in patients with mCRPC. Utilizing data from the Frankfurt Metastatic Cancer of the Prostate Database, the researchers analyzed 329 mCRPC patients who received [^177^Lu]Lu-PSMA therapy between the first and seventh lines of treatment. Among these, 19% (*n* = 64) had been pretreated with Ra-223, while 81% (*n* = 265) were Ra-223-naïve. The median interval between Ra-223 and [^177^Lu]Lu-PSMA treatments was 15 months.

Baseline characteristics, including age at mCRPC diagnosis (median: 71 years), PSA levels (median: 16 ng/mL), and Eastern Cooperative Oncology Group (ECOG) performance status (0 in 53%, 1 in 40%, and 2 in 6.8% of patients), were comparable between the two groups. However, Ra-223-pretreated patients had undergone a higher median number of systemic treatments for mCRPC (4 vs. 3; *p* < 0.01). Regarding treatment outcomes, the median PFS was 16.0 months for Ra-223-pretreated patients and 11.9 months for Ra-223-naïve patients (HR: 0.73; CI: 0.52–1.02; *p* = 0.063). The OS was 17.9 months for the pretreated group and 14.8 months for the naïve group (HR: 0.99; 95% CI: 0.71–1.37; *p* > 0.9). Multivariable analyses adjusting for factors such as age, Gleason score, ECOG status, and disease volume confirmed no significant differences in PFS and OS between the groups. These findings suggest that prior treatment with Ra-223 does not negatively impact the efficacy of [^177^Lu]Lu-PSMA, as both PFS and OS were comparable between the two groups. These results support the feasibility of administering [^177^Lu]Lu-PSMA therapy in patients previously treated with Ra-223 (Table 2).

#### 1.3.2. Actinium-225

Sgouros et al. [56] examined the dosimetric impact of ^227^Ac-contamination in accelerator-produced ^225^Ac (denoted ^225/7^Ac) used for alpha-emitter therapy targeting hematological malignancies. They modeled intravenous administration of ^225/7^Ac-conjugated anti-CD33 antibodies assuming a 0.7% activity of ^227^Ac at injection and a 70% retention of its daughter, ^227^Th, on the antibody. Key results show that ^227^Ac and its decay daughters deliver negligible absorbed doses, with maximum values of <0.02 mGy/MBq in organ tissues receiving the highest exposure (red marrow, spleen, endosteal bone surface, liver, lungs, kidneys) and <0.007 mGy/MBq in all other tissues.

Importantly, the cumulative dose contribution from ^227^Ac accounts for less than 0.04% of the total absorbed dose imparted by ^225^Ac and its daughters in these six highest-risk tissues. Across all examined tissues, ^227^Th is the primary dose contributor among ^227^Ac decay products. These results conclude that under the specified assumptions, contamination with ^227^Ac in accelerator-derived ^225^Ac produces clinically insignificant additional radiation dose to normal organs, affirming its potential use in hematologic alpha radiopharmaceutical therapy.

Rodak et al. [57] developed and preclinically characterized [^225^Ac]Ac-DOTA-2Rs15d, a HER2-targeted alpha radiotherapeutic based on a single-domain antibody (sdAb). Mass spectrometry confirmed efficient conjugation of one to two DOTA chelators per sdAb, and radiolabeling with actinium-225 achieved >90% yield and >98% purity. The compound showed strong HER2 binding (Kd = 3.50 ± 0.17 nM); receptor-specific internalization; and potent, dose-dependent cytotoxicity (EC_50_ = 3.9 ± 1.1 kBq/mL) with markedly increased DNA double-strand breaks. In SKOV-3 xenograft models, [^225^Ac]Ac-DOTA-2Rs15d demonstrated rapid, selective tumor uptake (peaking at 9.64 ± 1.69% injected activity per gram of tissue [IA/g]) at 3 h and stable retention, with minimal liver or bone accumulation and declining kidney uptake over time, indicating high tumor specificity and limited off-target exposure.

Therapeutic studies [57] showed significant survival benefits in SKOV-3-bearing mice, with a single 85 kBq dose extending median survival to 101 days and triple dosing extending it to143 days, compared with 56 days in controls. Combination with trastuzumab achieved the longest survival (161 days) but was not significantly superior to triple doses alone. Autoradiography confirmed uniform intratumoral distribution, while kidney analysis revealed dose-dependent tubular damage, highlighting the need for careful dosimetry to balance strong antitumor efficacy with renal safety. These findings support [^225^Ac]Ac-DOTA-2Rs15d as a promising HER2-targeted alpha therapy.

Bidkar et al. [58] developed a CD46-targeted alpha-particle therapy using the humanized antibody YS5 labeled with actinium-225. Biodistribution studies in 22Rv1 xenograft-bearing mice demonstrated high and sustained tumor uptake of [^225^Ac]Ac-DOTA-YS5, increasing from 11.6 ± 1.4 %ID/g at 24 h to 31.8 ± 5.9 %ID/g at 408 h, with favorable tumor-to-blood and tumor-to-muscle ratios. In vitro, [^225^Ac]Ac-DOTA-YS5 showed specific binding (IC_50_ = 3.0 ± 0.9 Bq/mL) and potent cytotoxicity in prostate cancer cell lines (e.g., 22Rv1 and DU145). Autoradiographic imaging and γ-H2AX staining confirmed effective tumor delivery and DNA damage induction (4.46–4.90 foci/cell vs. 1.37 in controls, *p* = 0.02).

Therapeutic studies in PSMA-positive (22Rv1) and PSMA-negative (DU145) xenografts, as well as in patient-derived xenograft (PDX) models (LTL-484, LTL-545), showed significant tumor suppression and survival benefit. In 22Rv1 mice, [^225^Ac]Ac-DOTA-YS5 at 9.3 and 18.5 kBq doses prolonged survival to 103 and 131 days, respectively, compared with 37 days in saline controls. A three-dose regimen (3 × 4.6 kBq) also enhanced efficacy with reduced toxicity. In LTL-545 PDX models, 4.6–9.3 kBq doses led to complete tumor regression with survival extended up to 198 days. However, nephrotoxicity was observed at higher doses (18.5–37.0 kBq), primarily due to 213Bi redistribution. Overall, [^225^Ac]Ac-DOTA-YS5 showed potent and selective antitumor activity across PSMA-positive and negative prostate cancer models, supporting its clinical translation potential.

Salvanou et al. [59] functionalized gold nanoparticles (AuNPs) with the chelator TADOTAGA that were radiolabeled with ^225^Ac to evaluate their potential for localized alpha therapy. The ultrasmall nanoparticles (2–3 nm core, 5–9 nm hydrodynamic diameter) were synthesized with high yield (86% ± 1.8%) and purity (>93%), showing excellent colloidal stability and ~80% retention of ^225^Ac over 10 days. In vitro studies in U-87 MG glioblastoma cells revealed potent dose- and time-dependent cytotoxicity, reducing cell viability by up to 87% after 48 h at 2 kBq/mL, while non-radioactive AuNPs were minimally toxic.

In vivo biodistribution showed limited tumor uptake after intravenous injection but high and sustained retention after intratumoral delivery (60.7 %IA/g at 2 h, 5.2% at 288 h), with minimal off-target accumulation. Therapeutic studies demonstrated significant tumor growth delay and extensive localized necrosis after three intratumoral injections (total 15 kBq/mouse), while sparing surrounding healthy tissue. These results highlight [^225^Ac]Ac-Au-TADOTAGA as a promising injectable nanobrachytherapy platform capable of delivering high-dose alpha radiation directly to tumors with minimal systemic toxicity.

Taddio et al. [60] evaluated the therapeutic potential of the alpha-emitting radioligand [^225^Ac]Ac-FAPI-46 in immunocompetent murine models of soft-tissue sarcoma, focusing on its efficacy as monotherapy and in combination with PD-1 immune checkpoint blockade (ICB). Fibroblast activation protein (FAP) is a theranostic target on cancer-associated fibroblasts and some sarcoma cells. [^225^Ac]Ac-FAPI-46 was synthesized with >97% radiochemical purity and a molar activity of ~45–55 MBq/µmol. In the parental FSA model, three cycles of 60 kBq [^225^Ac]Ac-FAPI-46 were well tolerated but had minimal impact on tumor growth. When combined with anti-PD-1 ICB in this model, 55% of mice (6/11) exhibited tumor growth delay, and 18% (2/11) showed partial tumor regression. These results indicate limited monotherapy efficacy, likely due to rapid tracer kinetics and low tumor retention inherent to FAPI-46. Furthermore, increasing tumoral FAP expression by generating FSA tumors overexpressing FAP (FSA-F) enhanced radioligand uptake and improved uptake metrics such as SUVmax (~3.2–3.44 vs. ~1.6 in parental tumors), correlating with increased therapeutic impact; however, in the FSA-F model both [^225^Ac]Ac-FAPI-46 and PD-1 ICB showed efficacy individually, and adding them together did not significantly enhance outcomes beyond PD-1 blockade alone.

To mimic an immunologically cold and ICB-resistant tumor microenvironment, the authors created a model by co-inoculating FSA-F cells with the immunosuppressive agent Abatacept, effectively abrogating PD-1 ICB responsiveness. In this setting, monotherapy with three injections of 60 kBq [^225^Ac]Ac-FAPI-46 or PD-1 ICB alone did not significantly improve survival relative to untreated controls (~28 days median survival), but the combination therapy restored ICB sensitivity, resulting in significant tumor regressions and durable tumor-free survival in 56% of mice (5/9) maintained out to at least 60 days post-treatment. This synergistic effect suggests that alpha-emitting FAP-targeted RLT can modulate the tumor immune microenvironment to overcome resistance to checkpoint inhibition. Overall, the findings underscore that the efficacy of [^225^Ac]Ac-FAPI-46 correlates with tumoral FAP expression levels and immune context, highlighting the need for biomarker-guided patient selection and rational combination strategies to maximize the therapeutic impact of FAP-targeted radioligands.

Khreish et al. [61] evaluated the safety and efficacy of tandem radioligand therapy combining low-dose alpha-emitter [^225^Ac]Ac-PSMA-617 with full-dose beta-emitter [^177^Lu]Lu-PSMA-617 in 20 heavily pretreated men with advanced mCRPC who had disease progression on [^177^Lu]Lu-PSMA-617 monotherapy. Patients received a median of 5.3 MBq [^225^Ac]Ac-PSMA-617 and 6.9 GBq [^177^Lu]Lu-PSMA-617. At 6–8 weeks post-therapy, 50% achieved a ≥50% PSA decline, 40% had stable disease, and 10% progressed. The best overall biochemical response was that 65% had ≥50% PSA reduction, and 90% experienced some PSA decline. Median PFS was 19 weeks, and median OS was 48 weeks.

Tandem therapy was generally well tolerated. Xerostomia was the most common side effect (grade 1 to 2 in 65%, no patient had grade ≥ 3), while hematologic toxicities (grade 3/4) were seen in 25% of patients, mostly in those with heavy prior treatment. Importantly, no grade ≥2 nephrotoxicity occurred. The approach appeared to preserve therapeutic benefit while reducing the risk of severe xerostomia associated with high dose ^225^Ac monotherapy. Subgroup analyses revealed no statistically significant differences in outcomes between patients with primary versus acquired resistance to [^177^Lu]Lu-PSMA-617. These results support further exploration of tandem ^225^Ac/^177^Lu-PSMA therapy in resistant mCRPC.

Besiroglu et al. [62] assessed the safety and efficacy of targeted alpha therapy using Ac-225-labeled prostate-specific membrane antigen ([^225^Ac]Ac-PSMA) in patients with mCRPC. Across 10 included studies with more than 300 patients, the pooled analysis showed that approximately 63% of patients achieved a ≥50% decline in PSA levels following treatment, with some cohorts reporting complete PSA response rates as high as 28%. Median PFS ranged from 6.8 to 15.15 months, while median OS varied between 9.52 to 15.91 months. Notably, patients who had previously failed multiple lines of therapy, including chemotherapy and Lutetium-177 PSMA therapy, still showed significant biochemical responses and clinical benefit from [^225^Ac]Ac-PSMA. The studies also highlighted that repeated treatment cycles (usually 2 to 4 cycles spaced six to eight weeks apart) correlated with improved tumor control and longer survival outcomes.

Regarding safety, the therapy demonstrated a manageable toxicity profile. The most frequent adverse event was xerostomia, reported in 62–72% of patients; however, it was mostly mild to moderate in severity. Grade 3 or higher hematologic toxicities such as anemia, thrombocytopenia, or leukopenia occurred in less than 10% of patients, with most events being transient and reversible. Severe nephrotoxicity or hepatotoxicity was rare (<5%). These findings suggest that [^225^Ac]Ac-PSMA is a potent therapeutic option with acceptable safety for heavily pretreated mCRPC patients. However, the analysis emphasized the need for larger randomized controlled trials to optimize dosing regimens, minimize side effects like xerostomia, and establish long-term efficacy when compared with other radionuclide therapies

Yadav et al. [63] evaluated the efficacy and safety of [^225^Ac]Ac-DOTATATE targeted alpha therapy in 10 patients with metastatic or inoperable paragangliomas who had progressive disease despite prior therapies. Patients received [^225^Ac]Ac-DOTATATE at administered activities of approximately 100 kBq/kg per cycle, given at 8–12 week intervals, for a median of 3 cycles (range 2–5). In the response assessment, partial response (PR) was observed in 4/10 patients (40%), and stable disease (SD) in 5/10 (50%), yielding a disease control rate of 90%. Only one patient (10%) developed progressive disease during follow-up. Biochemically, significant reductions in plasma or urinary catecholamine markers were observed in most evaluable patients. Clinically, improvement in tumor-related symptoms—including pain and catecholamine-excess manifestations—was reported in approximately 60–70% of patients. At a median follow-up of approximately 18 months, the median progression-free survival (PFS) had not been reached, and several patients maintained durable responses.

In terms of safety, the treatment was generally well-tolerated. Grade 1–2 hematologic toxicity (anemia, thrombocytopenia, or leukopenia) occurred in a minority of cycles, while grade 3 hematologic toxicity was observed in 1–2 patients, and no grade 4 events were reported. Importantly, no significant long-term nephrotoxicity was documented, and renal function remained stable in most patients with the use of standard amino acid–based nephroprotection. Xerostomia was mild and infrequent compared with that observed with PSMA-targeted alpha therapies. Although limited by a small sample size and the absence of a comparator arm, this study demonstrated a notably high disease control rate and a manageable toxicity profile in a heavily pretreated population, supporting ^2^[^225^Ac]Ac-DOTATATE as a promising therapeutic option for metastatic paragangliomas and providing a strong rationale for larger prospective trials.

Tagawa et al. [64] reported the first-in-human Phase I dose-escalation trial of the PSMA-targeted alpha-emitting radioimmunotherapy [^225^Ac]Ac-J591 in men with progressive mCRPC that was refractory to, or not eligible for, standard therapies. 32 patients were treated across seven predetermined dose cohorts ranging from 13.3 to 93.3 kBq/kg of [^225^Ac]Ac-J591 administered as a single IV dose. An accelerated escalation design was used initially with a single patient per dose level (levels 1–4), transitioning to a 3 + 3 schema for higher levels; dose-limiting toxicity (DLT) was monitored over the first 8 weeks. Only 1 of 22 patients (4.5%) in the escalation phase experienced a DLT at the 80 kBq/kg level (cohort 6), and no DLTs occurred at the highest dose (93.3 kBq/kg). Consequently, the maximum tolerated dose (MTD) was not reached, and the recommended Phase II dose (RP2D) was selected as 93.3 kBq/kg. Adverse events were predominantly hematologic, with non-hematologic toxicity largely low grade, reflecting a manageable safety profile in this heavily pretreated cohort. The median baseline PSA of treated patients was 149 ng/mL; most (78%) had received ≥2 androgen receptor pathway inhibitors, 63% had prior chemotherapy, and nearly half (47%) had prior [^177^Lu]Lu-PSMA therapy.

Efficacy signals were notable despite the single-dose design and lack of PSMA PET selection: 46.9% of patients experienced a ≥50% decline in PSA at any time, 34.4% had confirmed PSA responses, and 59.1% (13 of 22) achieved a protocol-defined circulating tumor cell (CTC) response. These biochemical responses were accompanied by declines in CTC counts (conversion to favorable or undetectable status), suggesting antitumor activity across dose levels. Importantly, the high-energy alpha emissions from ^225^Ac confer high linear energy transfer, enabling potent cytotoxicity over a short tissue range, potentially enhancing tumor kill while limiting off-target effects. The relatively low incidence of severe thrombocytopenia (grade 4, ~9.4% at RP2D) compared with historical ^177Lu-J591 data underscores a favorable hematologic toxicity profile with alpha therapy. These results support further investigation of ^225^Ac-J591—including fractionated and multiple-dose regimens now in follow-on Phase I/II studies—to more fully characterize its therapeutic index and clinical benefit in advanced mCRPC (Table 3).

#### 1.3.3. Bismuth-213

Havlena et al. [65] evaluated the therapeutic potential of ^213^Bi-labeled anti-CD20 monoclonal antibody (rituximab) for treating micrometastatic B-cell non-Hodgkin lymphoma (NHL) in a severe combined immunodeficiency mouse model. In vitro, ^213^Bi-rituximab produced strong, antigen-specific cytotoxicity causing complete cell death at 740 kBq/mL, while unlabeled or nonspecific controls showed minimal effects. An antigenic blockade with nonradiolabeled rituximab significantly reduced cytotoxicity, confirming target specificity despite the isotope’s short half-life (46 min). In vivo, a single 3700 kBq dose of [^213^Bi]Bi-rituximab given four days after tumor inoculation cured 75% of mice, outperforming [^90^Y]Y-rituximab and matching the efficacy of [^131^I]I-tositumomab. Two lower doses (2775 kBq each) achieved 67% potential cure, although efficacy declined with higher tumor burden. Histopathology confirmed minimal residual disease in treated animals. These results demonstrate that [^213^Bi]Bi-rituximab can achieve high cure rates in early-stage disseminated NHL, supporting alpha-emitter radioimmunotherapy (RIT) as a promising strategy for minimal residual disease and highlighting the potential of longer-lived isotopes like ^225^Ac for clinical translation.

Gustafsson et al. [66] evaluated the therapeutic efficacy and dose–response relationship of ^213^Bi-labeled monoclonal antibody MX35 ([^213^Bi]Bi-MX35) in treating microscopic ovarian cancer in a mouse model. MX35 targets the sodium-dependent phosphate transport protein NaPi2b, which is highly expressed in >90% of epithelial ovarian cancers. Female nude mice (*n* = 60) were injected intraperitoneally with ~1 × 10^7^ OVCAR-3 human ovarian cancer cells to mimic microscopic intraperitoneal (IP) disease. Two weeks post-tumor inoculation, 40 mice received a single IP injection of [^213^Bi]Bi-MX35 at activity levels of 9 MBq/mL (group 1, 20 mice) and 3 MBq/mL (group 2, 20 mice). An untreated control group was included by receiving 30 μg unlabeled MX35 (20 mice).

At eight weeks of post-treatment, all mice were euthanized and evaluated for macroscopic tumor burden. Tumor-free fractions were 0.15 in the control group, 0.55 at 3.0 MBq, and 0.78 at 9.0 MBq. A clear dose–response was observed: higher administered activity levels significantly improved therapeutic efficacy (*p* < 0.05). The treatment was well tolerated with no significant weight loss or observable toxicity in any group. Biodistribution data and earlier studies indicated that the short range and high energy of ^213^Bi alpha emissions enabled effective local tumor cell killing with minimal collateral damage to surrounding healthy tissues. This study supports the potential of IP alpha-RIT with ^213^Bi-MX35 as an effective treatment for microscopic ovarian cancer. Importantly, the results indicate that higher activity levels substantially increase tumor eradication rates, providing critical data for dose optimization in future clinical translation efforts.

Deshayes et al. [67] designed and characterized 16F12, a murine monoclonal antibody targeting MISRII that is expressed in ~70% of epithelial ovarian cancers, and conjugated it with ^89^Zr for positron emission tomography (PET) imaging, ^177^Lu (β-emitter), or ^213^Bi (alpha-emitter) for therapy. In a peritoneal xenograft model, IP-RIT with 10 MBq [^177^Lu]Lu-16F12 delayed tumor growth more effectively than 12.9 MBq [^213^Bi]Bi-16F12, but brief IP-RIT (BIP-RIT) reversed this trend: 37 MBq [^213^Bi]Bi-16F12 reduced mean tumor mass to 0.012 g by day 30, with tumor-to-blood dose ratios improving from 1.4 to 6, indicating enhanced tumor selectivity.

Safety profiles favored alpha-therapy, as hematologic toxicity was far lower with [^213^Bi]Bi-16F12 (blood ~1.6 Gy) than with [^177^Lu]Lu-16F12 (blood ~13.5 Gy). Imaging confirmed targeted antibody uptake with both SPECT (^177^Lu) and PET (^89^Zr). These findings support a theranostic strategy using 16F12 where PET imaging guides alpha-RIT and with BIP-RIT delivering potent tumor control and minimal systemic toxicity, which is particularly promising for adjuvant treatment after cytoreductive surgery.

Ladjohounlou et al. [68] investigated how drugs that alter cholesterol metabolism influence both targeted and nontargeted cellular responses during alpha-particle (^212^Pb/^212^Bi, ^213^Bi) and Auger (125I) RIT in ovarian cancer models. In vitro results showed that alpha-RIT induced targeted cell death in 67–94% of cancer cells, while Auger-RIT killed only 8–15% directly; however, nontargeted (bystander) effects were substantial: 7–36% for alpha-RIT and 27–29% for Auger-RIT. Mechanistic studies revealed that these effects were partly mediated through lipid-raft dependent activation of stress kinases p38 and JNK and reactive oxygen species-driven NF κB activation

When cells were co-treated with cholesterol-modifying agents—like the acid sphingomyelinase inhibitor imipramine or lipid-raft disruptors (methyl beta cyclodextrin or filipin)—there was a significant reduction in nontargeted cytotoxicity. This led to increased clonogenic survival in vitro. Moreover, pravastatin (a statin) also reduced Auger RIT efficacy. In vivo, xenografted mice receiving RIT plus these cholesterol-targeting drugs exhibited larger tumor volumes and less DNA damage in normal tissues when compared with those receiving RIT alone, underscoring that nontargeted membrane-based signaling is crucial for RIT’s full therapeutic effect. The findings reveal that both direct and indirect (bystander) mechanisms play major roles in the effectiveness of alpha- and Auger-RIT, with lipid rafts and cholesterol metabolism mediating p38/JNK and ROS signaling that amplifies treatment efficacy. Consequently, incorporating cholesterol-modifying drugs like statins may inadvertently diminish RIT effectiveness by suppressing these nontargeted pathways. These insights suggest that clinical strategies combining RIT with other agents must carefully consider cholesterol metabolism’s modulation of p38/JNK driven responses to optimize therapeutic outcomes.

Jiao et al. [69] used a syngeneic Cloudman S91 melanoma model in DBA/2 mice to evaluate whether combining alpha particle RIT targeting melanin with anti-programmed death-1 (PD-1) immunotherapy could improve outcomes versus single-modality treatment. Male and female DBA/2 mice were subcutaneously implanted with 3–6 × 10^6^ tumor cells and treated once tumors reached ~150 mm^3^. Groups received either phosphate-buffered saline (PBS), 80 µg unlabeled melanin-targeting h8C3 antibody (cold), three doses (250 μg) of anti-PD 1 mAb (Days 8, 11, 14), a single dose of 14.8 MBq ^213^Bi labeled h8C3 (Day 8), or combinations in various sequences either anti-PD 1 first followed by RIT or RIT followed by anti-PD 1. Single-agent therapies modestly slowed tumor growth, while the optimal schedule anti-PD 1 “sandwiched” between two 14.8 MBq RIT doses on days 8 and 15 significantly delayed progression: time to reach 1500 mm^3^ increased from ~26–31 days for single modalities to ~43.5 days for combination therapy.

In terms of survival and toxicity, mice receiving the dual treatment of two RIT doses plus anti-PD 1 showed the longest survival and exhibited minimal systemic toxicities; body weight transiently dipped after RIT but recovered fully by day 22 with continued weight gain. Immunophenotyping confirmed increased CD8^+^ T-cell infiltration in the tumor microenvironment in combination-treated animals, highlighting potential immune priming by RIT. The study demonstrates that alpha particle RIT combined with anti-PD 1 therapy produces synergistic antitumor effects delaying tumor growth by 1.5 times, extending survival, and enhancing CD8^+^ T-cell tumor infiltration with no additional toxicity in this immunocompetent model. These promising results advocate for further exploration of RIT-immunotherapy combinations in melanoma and potentially other immunogenic cancers.

Krolicki et al. [70] investigated [^213^Bi]Bi-DOTA-substance P (SP)-targeted alpha therapy in nine patients with recurrent secondary glioblastoma multiforme. Patients received one to six intracavitary or intratumoral injections (median total activity 5.8 GBq) at two-month intervals, with [^68^Ga]Ga-DOTA-SP used for PET/CT monitoring. The treatment was well tolerated, causing only mild, transient side effects, and it showed minimal systemic exposure (<8% blood activity, <5% urinary excretion), confirming strong tumor localization.

Median progression-free survival was 5.8 months, and median overall survival from therapy start was 16.4 months, with two patients surviving beyond 39 and 51 months despite prior surgery, radiotherapy, and chemotherapy. Magnetic resonance imaging showed moderate, although not statistically significant, tumor volume reductions. Compared with typical survival of less than 10 months for similar patients, these results highlight [^213^Bi]Bi-DOTA-SP as a safe, locally administered alpha therapy with encouraging efficacy, supporting further trials and development of scalable ^225^Ac/^213^Bi production (Table 4).

#### 1.3.4. Bismuth-212

Kauffman et al. [71] evaluated the therapeutic efficacy of Bismuth-212-labeled macroaggregated albumin ([^212^Bi]Bi-MAA) in orthotopic mouse breast tumor models, aiming to leverage alpha-particle therapy for localized radiation delivery. ^212^Bi, an alpha emitter, was eluted from a ^212^Pb generator and bound to MAA with a radiolabeling efficiency of ~50% (e.g., 17.9 ± 0.1 MBq Bi-212 yielded 8.9 ± 0.04 MBq ([^212^Bi]Bi-MAA). Clonogenic and survival assays in vitro demonstrated that doses as low as 92.5 kBq significantly reduced colony formation and cell survival in both 4T1 and EO771 murine breast cancer cell lines. Western blot analysis further revealed increased γH2AX (a marker of DNA damage response) and cleaved caspase-3 expression in ([^212^Bi]Bi-MAA-treated cells, indicating DNA damage and apoptosis. At higher doses, checkpoint markers (Chk1, Chk2, Wee1) were not activated, suggesting cell death without induction of cell cycle arrest—an advantage in avoiding radioresistance.

In vivo studies using intratumoral injection showed high retention of ([^212^Bi]Bi-MAA within tumors: 87–93% of the injected dose remained localized at both 2 and 4 h post-injection, minimizing systemic exposure. Tumor growth was significantly inhibited in both 4T1 and EO771 models following a single injection of ([^212^Bi]Bi-MAA (925–3700 kBq). For instance, 1850–3700 kBq in EO771 tumors and 34–68 MBq in 4T1 tumors led to substantial reduction in tumor volume over an eight-day period when compared with controls. Cherenkov and bioluminescence imaging confirmed co-localization of the radiotracer with tumor cells, supporting effective delivery. This preclinical study demonstrates that ([^212^Bi]Bi-MAA is a promising, rapidly translatable alpha-particle radiotherapeutic for solid tumors, with potential for intra-arterial delivery in human settings using existing FDA-approved components.

Wang et al. [72] presents a novel in vivo alpha-particle generator system based on ultrasmall glutathione-coated silver telluride nanoparticles (GSH-Ag_2_TeNPs) radiolabeled with lead-212 (^212^Pb), aiming to improve targeted radionuclide therapy by enhancing daughter nuclide retention. The nanoparticles had a core diameter of 2.1 ± 0.3 nm and a hydrodynamic diameter of 2.5 ± 0.1 nm, favoring renal clearance. The ^212^Pb radiolabeling efficiency reached 75%, and radiochemical stability remained exceptionally high: 103 ± 5% at 4 h and 96 ± 6% at 24 h post-incubation with ethylenediaminetetraacetic acid. This indicates excellent retention of both ^212^Pb and its daughter ^212^Bi, overcoming a major limitation of conventional DOTA-based chelators, which lose up to 37% of ^212^Bi due to internal conversion effects.

To assess biological functionality, the nanoparticles were also radiolabeled with ^111^In for in vitro evaluation. Uptake studies in U87 glioblastoma cells revealed nuclear accumulation of approximately 25% of internalized [^111^In]In-DTPA-Ag_2_TeNPs. Confocal microscopy confirmed nuclear localization. In a clonogenic survival assay, [^111^In]In-DTPA-Ag_2_TeNPs at 2 and 3 MBq induced significant tumor cell killing (*p* < 0.001 and *p* < 0.0001, respectively), compared with only 15% reduction with [^111^In]In-DTPA alone. These results were attributed to the Auger electron emissions of ^111^In when localized in the nucleus, supporting the therapeutic potential of similar ^212^Pb-loaded constructs.

In conclusion, the study establishes GSH-Ag_2_TeNPs as promising carriers for ^212^Pb/^212^Bi in vivo generators, offering superior radionuclide retention, nuclear delivery, and cytotoxic potential. Their small size enables renal clearance and intracellular penetration, making them ideal for development of theranostic agents combining PET/SPECT imaging (^203^Pb/^111^In) and alpha therapy (^212^Pb). Future work will involve functionalization with tumor-targeting ligands for specific cancer applications (Table 5).

#### 1.3.5. Astatin-211

Huang et al. [73] developed a novel targeted alpha-particle therapy (TAT) system based on ^211^At-labeled gold nanoparticles (AuNPs) for intravenous administration in a pancreatic cancer model. Four types of AuNPs were synthesized, with the 5 nm AuNPs modified by methoxy-polyethylene glycol (mPEG) showing the most favorable properties. These 5 nm [^211^At]At-AuNPs-mPEG demonstrated high radiochemical yields (>90% within 5 min), stability, and passive tumor accumulation via the enhanced permeability and retention (EPR) effect. Biodistribution data revealed that 5 nm [^211^At]At-AuNPs-mPEG accumulated in tumor tissue at 2.25 ± 0.67 %ID/g at 3 h and 1.80 ± 0.20 %ID/g at 24 h—significantly higher than the 30 nm counterparts. Importantly, the 5 nm particles cleared more rapidly from non-target organs, reducing off-target toxicity.

Therapeutic studies in PANC-1 tumor-bearing mice showed that a single intravenous injection of [^211^At]At-AuNPs-mPEG with a size of 5 nm at 1 MBq significantly suppressed tumor growth over 30 days when compared with controls. Tumor weights were markedly reduced (*p* < 0.01), and no severe toxicity was observed; body weight briefly declined but recovered within two weeks. While peptide-modified AuNPs (e.g., H16 or H16/RGD) showed higher cellular uptake and induced more DNA double-strand breaks (γH2AX expression) in vitro, they resulted in elevated liver accumulation in vivo, making them less suitable for systemic delivery. Conversely, mPEG-modified AuNPs showed balanced biodistribution and safety profiles. This study demonstrated effective tumor suppression using systemically delivered 5 nm ^211^At-labeled AuNPs, introducing a promising platform for targeted alpha therapy in metastatic cancers. The nanoparticles’ small size, efficient ^211^At-labeling, tumor selectivity, and minimal systemic toxicity make them attractive for future clinical development. Further optimization of targeting ligands and surface modifications could enhance delivery and therapeutic index even more.

Kato et al. [74] evaluated a novel intratumorally administered alpha-nanoparticle system using ^211^At-labeled gold nanoparticles (AuNP-S-mPEG) with various particle sizes (5, 13, 30, and 120 nm). The study was conducted on subcutaneous C6 glioma (rat) and PANC-1 pancreatic cancer (mouse) tumor models. After single intratumoral injections (~1.4 MBq for rats, ~1.2 MBq for mice), scintigraphy and autoradiography confirmed that ^211^At remained highly localized in tumors for up to 42 h (∼6 half-lives) with no detectable systemic leakage. Tumor growth suppression was significantly greater in the 5 nm group, with tumor weights reduced significantly when compared with saline controls (*p* < 0.001). The therapeutic efficacy was size-dependent, with smaller nanoparticles exhibiting better intratumoral diffusion and cytotoxicity.

In vitro cytotoxicity assays revealed that only the 120 nm [^211^At]At-AuNP-S-mPEG caused significant cell death at 1 MBq/mL, likely due to sedimentation-enhanced uptake. However, in vivo, smaller nanoparticles (5–30 nm) demonstrated superior tumor uptake and therapeutic outcomes. Autoradiographic analysis confirmed that 30 nm particles spread more evenly throughout tumor tissues, whereas 120 nm particles aggregated locally, leading to heterogeneous radiation exposure. Across all groups, no significant weight loss or off-target toxicity was observed in animals, supporting the safety of the approach. Importantly, unlabeled AuNP-S-mPEG showed no cytotoxicity in either in vitro or in vivo models. This study highlights the potential of non-targeted [^211^At]At-AuNP-S-mPEG as an effective local alpha-radiation therapy for solid tumors. The nanoparticles remained confined to the tumor site and delivered strong cytotoxic effects without damaging healthy tissues. The results support further development of size-optimized nanoseeds (5–30 nm) for outpatient brachytherapy applications using clinically compatible components like polyethylene glycol (PEG) and gold. The simplicity of ^211^At labeling and the favorable retention profile suggest clinical translation potential for various tumor types

Bäck et al. [75] evaluated systemic targeted alpha therapy using an ^211^At-labeled anti-PSCA A11 minibody in two prostate cancer models: PC3 cells were implanted in male nude mice with 100,000 cells in group 2, five times more than in group 1. Therapy was administered in two intravenous fractions spaced 14 days apart, using activity levels of 1.5 and 1.9 MBq for macrotumors and 0.8 or 1.5 MBq for microtumors. In macrotumor-bearing mice, the treated group exhibited an approximate 42% reduction in mean tumor volume at week 5 when compared with controls. Hematological monitoring revealed transient white blood cell decreases at day 6, which recovered by day 13 for the 0.8 and 1.5 MBq cohorts; a higher 2.4 MBq dose produced overt toxicity, requiring euthanasia by day 7.

In the microtumor model (~100–200 µm lesions), efficacy was assessed over two experiments. Experiment 1 showed a 95% TFF in treated animals versus 66% in controls (*p* < 0.05). In experiment 2, TFFs were 32% for treated and 20% for control groups, yet the treated cohort displayed a mean microtumor volume of 0.010 ± 0.003 mm^3^ compared with 3.79 ± 1.24 mm^3^ in controls, a 99.7% volumetric reduction (*p* < 0.001). Notably, long-term follow-up to day 252 showed normalized blood counts and no radiotoxicity in the 0.8 and 1.5 MBq groups, despite some body-weight loss between days 30–90. These results demonstrate potent, dose-dependent antitumor activity and durable safety of ^211^At-A11 minibody therapy, supporting further development of PSCA-targeted alpha-RIT for early metastatic prostate cancer.

Echigo et al. [76] presents the development and evaluation of a novel albumin-binding arginyl-glycyl-aspartic acid peptide radiolabeled with astatine-211, named [^211^At]5, for TAT. Incorporation of a 4-(4-astatophenyl)butyric acid (APBA) moiety—an analog of the known albumin-binding compound 4-(p-iodophenyl)butyric acid—prolonged blood circulation, enhancing tumor uptake and retention. In biodistribution studies using U-87 MG tumor-bearing mice, [^211^At]5 showed significantly higher tumor accumulation (10.42 ± 0.40 %ID/g at 1 h) when compared with a non-albumin binding moiety counterpart [^211^At]2 (7.28 ± 0.73 %ID/g) and much higher than the non-albumin binding [^67^Ga]3 (1.84 ± 0.09% ID/g at 24 h). [^125^I]4 and [^211^At]5 both demonstrated prolonged blood retention and tumor localization versus non-(albumin binding moiety) ABM tracers, confirming APBA’s functionality as an albumin-binding moiety

Therapeutic studies in tumor-bearing mice showed that [^211^At]5 significantly inhibited tumor growth in a dose-dependent manner. Mice treated with 925 kBq of [^211^At]5 exhibited greater tumor suppression than those receiving 370 kBq or vehicle control (*p* < 0.01). No marked hematotoxicity or weight loss was observed, even at the higher dose of 1.30 MBq used in toxicity assessments, suggesting minimal side effects. Furthermore, [^211^At]5 demonstrated high in vitro and in vivo stability, with >97% and 69% for radiochemical purity and radiochemical yield, respectively, retained after 24 h in PBS and minimal deastatination in vivo. These findings support the design of albumin-binding alpha radiopharmaceuticals as a promising strategy to harness the full therapeutic potential of ^211^At in targeted alpha therapy (Table 6).

#### 1.3.6. Lead-212

Stallons et al. [77] investigated the [^212^Pb]Pb DOTAMTATE, a somatostatin receptor targeted peptide, labeled with alpha-emitting ^212^Pb that showed robust tumor delivery and therapeutic potential in somatostatin receptor 2 (SSTR2)-positive neuroendocrine tumor models. Biodistribution studies in tumor-bearing mice revealed tumor uptake exceeding 20 %ID/g sustained through 24 h post-injection. Importantly, co administration of kidney-protective amino acids (L lysine/L arginine) successfully reduced renal exposure, while inclusion of ascorbic acid during labeling minimized peptide oxidation and improved tumor binding.

Safety assessments demonstrated excellent tolerability: a single 740 kBq dose led to 100% survival with only mild, reversible weight and leukocyte changes. Fractionated dosing up to 1665 kBq (three cycles, each 555 kBq) remained non-toxic; the no-observed-adverse-effect level was 370 kBq, and the highest non-severely toxic dose was 740 kBq. Efficacy studies were equally promising: one 370 kBq injection extended median survival 2.4-fold, and three cycles at biweekly intervals of 370 kBq each achieved complete tumor responses in ~50% of mice. Adding low-dose 5 fluorouracil further boosted outcomes: after three cycles, 79% of animals remained tumor-free at 31 weeks. Collectively, [^212^Pb]Pb DOTAMTATE demonstrates high tumor targeting (>20 %ID/g), a favorable safety window (≤1665 kBq fractionated), and potent antitumor activity complete cures in up to ~80% of mice when combined with chemo sensitizers. These results support its translation to clinical trials for SSTR positive neuroendocrine tumors.

Kasten et al. [78] evaluated the human monoclonal antibody 225.28, which binds chondroitin sulfate proteoglycan 4 (CSPG4), a proteoglycan overexpressed in ~73% of primary triple-negative breast cancers (TNBC). The antibody was labeled with lead-212 (^212^Pb) to enable alpha particle RIT in mouse models. In vitro assays demonstrated high-affinity binding (Kd ≈ 0.5 nM) to both adherent TNBC cell lines (SUM159, 2LMP) and cancer initiating cell (CIC) mammospheres, with ~10 fold more binding sites on SUM159 than on 2LMP. Clonogenic survival studies showed [^212^Pb]Pb 225.28 was six to seven times more effective at cell killing than a non-targeted isotype control ([^212^Pb]Pb F3 C25) in SUM159 cells and CICs (*p* < 0.05). Biodistribution in SUM159 and 2LMP xenografts revealed significantly higher tumor uptake of [^212^Pb]Pb 225.28 versus 125I- or 99mTc labeled control mAb (*p* < 0.05).

In SUM159-bearing mice, a single intravenous dose of 0.30 MBq of [^212^Pb]Pb 225.28 significantly inhibited tumor growth compared with 0.33 MBq of [^212^Pb]Pb F3 C25 (*p* < 0.01). The therapeutic effect was dose-dependent, with higher tumor control achieved at 0.30 MBq. These results demonstrate that targeting the CSPG4 epitope with [^212^Pb]Pb 225.28 yields potent antitumor responses in CSPG4-expressing TNBC models. Collectively, the study supports further development of [^212^Pb]Pb 225.28 as a promising RIT candidate for CSPG4-positive TNBC.

Kasten et al. [79] demonstrated the efficacy of the alpha-particle-emitting radioimmunoconjugate [^212^Pb]Pb-376.96, which targets the tumor-associated antigen B7-H3, in preclinical models of pancreatic ductal adenocarcinoma (PDAC). The antibody showed high-affinity binding to both differentiated PDAC3 cells (Kd ≈ 9.0 nM) and CICs from tumorspheres (Kd ≈ 21.7 nM) with significant binding site density. In vitro, [^212^Pb]Pb-376.96 inhibited clonogenic survival of both cell populations three to six times more effectively than an isotype-matched control ([^212^Pb]Pb-F3-C25). This enhanced efficacy was attributed in part to the compact nature of CIC tumorspheres, allowing effective traversal by alpha-particles and the higher expression of B7-H3 in CICs.

In vivo studies in mice bearing subcutaneous Panc039 xenografts and orthotopic PDAC3 tumors confirmed targeted uptake of [^212^Pb]Pb-376.96 with tumor accumulation significantly higher than controls (14.0 ± 2.1 %ID/g vs. 6.5 ± 0.9 %ID/g, *p* < 0.001). A single intravenous dose of [^212^Pb]Pb-376.96 (0.36–0.73 MBq) significantly inhibited tumor growth compared with controls, with higher doses producing greater effects. The treatment was well tolerated with only transient weight loss and no overt toxicity. However, no complete tumor regressions were observed, highlighting the challenge of eradicating established solid PDAC tumors. These findings support the potential of [^212^Pb]Pb-376.96 for B7-H3-targeted alpha-radioimmunotherapy and suggest future work should include fractionated dosing and a combination with chemotherapy to enhance efficacy.

Maaland et al. [80] investigated the therapeutic efficacy and safety of the alpha-emitting radioimmunoconjugate [^212^Pb]Pb-NNV003, which targets the B-cell antigen CD37, in preclinical models of chronic lymphocytic leukemia (CLL) and NHL. [^212^Pb]Pb-NNV003 demonstrated a strong in vitro cytotoxic effect in both MEC-2 (CLL) and Daudi (Burkitt’s lymphoma) cells, with Daudi cells showing greater sensitivity due to higher CD37 expression. In vivo biodistribution studies showed that [^212^Pb]Pb-NNV003 localized effectively in tumors—reaching 23 %ID/g in Daudi and 16 %ID/g in MEC-2 xenografts—without significant off-target accumulation, indicating stability and specificity.

In therapeutic studies, a single intravenous injection of [^212^Pb]Pb-NNV003 significantly improved survival in both models. In the Daudi model, 91% of mice survived 28 weeks after treatment with just 90 kBq, while MEC-2-bearing mice required higher doses (≥370 kBq) for similar benefit, reflecting their more aggressive tumor profile. Importantly, hematological toxicity was modest and dose-dependent; platelet counts dropped transiently at higher doses but recovered, and red and white blood cell counts remained stable. The highest non-severely toxic dose was defined as 555 kBq in R2G2 mice. The study concluded that [^212^Pb]Pb-NNV003 exhibits potent anti-tumor effects with manageable toxicity and supports its further development for clinical use in CD37-positive hematological malignancies.

Meredith et al. [81] evaluated the safety and potential biomarkers associated with IP administration of the alpha-emitting radioimmunotherapy agent [^212^Pb]Pb-TCMC-trastuzumab in 18 patients with HER2-expressing peritoneal metastases primarily from ovarian cancer in the first-in-human phase 1 study. Patients received a single IP dose at escalating levels (7.4 to 27.4 MBq/m^2^) following an intravenous pre-dose of trastuzumab. The treatment was well tolerated across all dose levels, with only mild and transient grade 1 adverse events, including liver enzyme fluctuations and asymptomatic hematologic changes. Importantly, no late toxicities in the renal, liver, hematologic, or cardiac systems were observed over one year, and no immune responses to the radioconjugate were detected in any of the 15 tested patients.

Tumor marker analysis revealed that TAG-72 (CA72-4) had the strongest correlation with clinical outcomes, showing up to a 66% decrease in levels six weeks post-treatment and a correlation coefficient (R^2^) of 0.73 with increasing radioactivity, outperforming computed tomography (CT) imaging (R^2^ = 0.21). Other markers like carbohydrate antigen (CA125), human epididymis protein 4 (HE-4), interleukin-6 (IL-6), mesothelin, serum amyloid A (SAA), and carcinoembryonic antigen (CEA) did not consistently correlate with imaging or therapeutic outcomes. Despite all patients eventually progressing within eight months, many lived longer than a year, enabling extended safety monitoring. The favorable safety profile and preliminary efficacy suggest that [^212^Pb]Pb-TCMC-trastuzumab is a promising candidate for further clinical development, especially in combination or adjuvant settings for microscopic residual disease (Table 7).

#### 1.3.7. Terbium-149

Umbricht et al. [82] achieved successful synthesis of [^149^Tb]Tb PSMA 617 with high radiochemical purity (>98%) at approximately 6 MBq/nmol and administered it to mice bearing PSMA-positive PC 3 PIP xenografts. Three dosing protocols were tested: a single 6 MBq injection on day 0, and two split-dose regimens of 3 MBq on days 0 + 1 or days 0 + 3. All treated groups showed significant tumor growth delay compared with controls (*p* < 0.05), with the two-dose (days 0 + 1) regimen being most effective. Median survival increased to 36 days in treated mice from 20 days in untreated mice.

Beyond therapeutic effects, ^149^Tb emits positrons, enabling PET/CT imaging of tumor uptake. Imaging at 0.5–4 h post-injection confirmed selective accumulation in PSMA-positive tumors, demonstrating the feasibility of “alpha PET” for simultaneous treatment and real-time monitoring of biodistribution. Dosimetry estimates showed tumor and kidney absorbed doses of ~6.9 Sv/MBq and ~0.63 Sv/MBq, respectively, when considering a factor of five for the RBE of the alpha radionuclide. The tumor dosimetry is roughly doubled when compared with [^177^Lu]Lu PSMA 617, with acceptable kidney exposure. The short half-life of ^149^Tb also improved tumor-to-background dose ratios when compared with 213Bi. These findings highlight that [^149^Tb]Tb PSMA 617 provides potent alpha-particle therapy with PET guidance, although limited radionuclide supply and extreme PSMA overexpression constrain complete tumor eradication. Further studies should refine dosing, explore combinations, and scale production for clinical translation.

Muller et al. [83] presented a compelling case for ^149^Tb as a dual-purpose radionuclide, combining short-range alpha-particle therapy with PET imaging capabilities. The physical characteristics of a half-life of ~4.1 h, moderate alpha-energy (~3.97 MeV, LET ~140 keV/µm), and absence of alpha-emitting daughters render ^149^Tb well-suited for targeted radiotherapy with limited off-target toxicity. Crucially, its decay also emits positrons (mean Eβ+ ≈ 730 keV, β+ yield ~7.1%), enabling PET imaging, plus a γ line (~165 keV) suitable for single-photon emission computed tomography (SPECT). These properties, combined with stable complexation using DOTA chelators, position ^149^Tb as a versatile agent in radiotheranostic approaches.

The authors [83] validate these advantages by showcasing a preclinical PET/CT study in mice bearing AR42J tumors labeled with [^149^Tb]Tb-DOTANOC (~7 MBq). Images acquired 2 h post-injection displayed high tumor accumulation with low background uptake in kidneys and bladder—demonstrating proof of concept for “alpha-PET” imaging. This experiment illustrates the potential to monitor therapeutic biodistribution in real time, guiding dose planning and safety evaluation. While not addressing therapeutic efficacy directly, the study builds upon prior data showing cancer cell killing in receptor-targeted folate models using ^149^Tb, highlighting its potent alpha-therapeutic potential. Overall, ^149^Tb combines effective alpha therapy with intrinsic PET capability, warranting further development in isotope production, targeting of vectors, and advanced therapeutic studies for future clinical translation (Table 8).

#### 1.3.8. Thorium-227

Lejeune et al. [84] evaluated the mesothelin-targeted thorium 227 conjugates (MSLN TTC) in activated innate immune pathways in mesothelin-expressing cancer cell lines. Transcriptomic profiling revealed upregulation of DNA sensing and type-I interferon genes (IL 6, CCL20, CXCL10, STING-related), confirmed by detection of phosphorylated stimulator of interferon genes (STING) protein. Concurrently, damage-associated molecular patterns (DAMPs) were increased, which led to dendritic cell activation in co-culture assays.

In immunocompetent MC38 hMSLN tumor-bearing mice, MSLN TTC monotherapy significantly inhibited tumor growth (tumor/control (T/C) ~0.38, *p* < 0.05). When combined with anti PD L1, the antitumor effect was markedly enhanced (T/C ~0.08, *p* < 0.001), resulting in more tumor-free survivors. Crucially, depleting CD8^+^ T cells diminished the therapeutic effect, underscoring the role of adaptive immunity. This work demonstrates that TTCs kill tumor cells and stimulate STING-driven immunity with strong synergy alongside PD-L1 blockade, supporting clinical evaluation of MSLN-TTC plus checkpoint inhibitors.

Berg-Larsen et al. [85] explored the dual therapeutic and immunostimulatory potential of targeted TTCs in the syngeneic MC-38 murine colorectal cancer model. In vitro, both isotype and PD L1-targeted TTCs induced robust DNA double-strand breaks and immunogenic cell death in MC-38 cells evidenced by γH2AX formation and increased expression of DAMPs such as HMGB1, CRT, and heat shock proteins (HSPs). Ex vivo, mice implanted with MC-38 cells pre-treated with TTCs demonstrated enduring tumor-specific immunity: nine of 10 mice resisted tumor growth upon reinoculation with live MC-38 cells, while the control animals developed tumors rapidly.

In vivo efficacy experiments revealed that a single systemic dose of 500 kBq/kg TTC significantly inhibited MC-38 tumor growth when compared with vehicle controls. When combined with an anti-PD 1 checkpoint blockade (10 mg/kg, twice weekly), tumor suppression was markedly enhanced. Notably, mono treated mice displayed delayed tumor recurrence (~60 days), whereas the combination group exhibited no tumor regrowth, indicative of durable tumor control. Subsequent rechallenge experiments confirmed robust, antigen-specific immune memory: combination-treated mice resisted MC-38 rechallenge but allowed growth of unrelated B16-F10 tumors. Immune profiling further demonstrated significant infiltration of CD8^+^ T cells and mature dendritic cells in treated tumors, reinforcing the pivotal role of adaptive immunity in mediating the observed tumor eradication. In summary, TTCs deliver precise alpha-therapy while stimulating adaptive immunity, and combined PD-1 blockade achieves long-term tumor eradication—supporting clinical testing of TTC + checkpoint inhibitor strategies.

Hagemann et al. [86] reported a mesothelin-targeted thorium-227 conjugate (MSLN-TTC), BAY 2287411, as a novel TAT with potent and selective antitumor effects against MSLN-expressing solid tumors such as mesothelioma and ovarian, pancreatic, lung, breast, and colorectal cancers. This fully human IgG1 antibody (BAY 86-1903) conjugated to an octadentate 3,2 hydroxypyridinone (3,2-HOPO) chelator and labeled with thorium-227 showed stable binding and specific radiolabeling. In vitro studies across 12 MSLN-positive cell lines demonstrated that BAY 2287411 induces double-strand DNA breaks (via γ-H2AX), oxidative stress, G2-M arrest, apoptosis (caspase-3/7 activity), and necrosis, with cytotoxicity correlating with MSLN expression levels. Remarkably, it also upregulated DAMPs, suggesting potential immunogenic cell death, which may enable synergy with immune checkpoint inhibitors. Enhanced sensitivity was noted in BRCA1-deficient models, hinting at potential combinatory use with DNA repair inhibitors.

In vivo studies using both cell line-derived (CDX) and patient-derived xenograft (PDX) models demonstrated strong tumor accumulation and retention of BAY 2287411, particularly in high MSLN-expressing tumors. A single 500 kBq/kg dose yielded tumor regression in HT29-MSLN (T/C: 0.09), ST103 (T/C: 0.02), and ST2185B (T/C: 0.1) models. Even tumors with only 10–20% MSLN-positive cells responded due to the alpha emitter’s short but effective path length (2–10 cell diameters), indicating a crossfire effect. In a disseminated lung cancer model (NCI-H226-luc), BAY 2287411 improved median survival by up to 84 days. Importantly, it was well-tolerated, with only reversible leukopenia as an adverse effect. Pharmacokinetic and pharmacodynamic models confirmed that efficacy correlates with tumor doubling time and MSLN density, and an optimal antibody dose (0.14 mg/kg) can ensure effective tumor cell hits (>8–36/cell). These comprehensive preclinical data supported the initiation of a phase 1 clinical trial in mesothelioma and ovarian cancer.

Karlsson et al. [87] presented thorium-227 conjugates (TTCs) as a promising class of radiopharmaceuticals for alpha-particle therapy, offering precise and potent cytotoxicity to tumor cells with minimal damage to surrounding healthy tissues. These agents consist of thorium-227, a high LET alpha-emitter stably chelated via a 3,2-HOPO ligand to antibodies; peptides; or small molecules targeting tumor-associated antigens, such as HER2, PSMA, CD70, CD22, mesothelin, and FGFR2. Preclinical studies across various cancer models, including breast, prostate, ovarian, lymphoma, and mesothelioma, have demonstrated significant anti-tumor efficacy even in resistant or metastatic settings. Notably, HER2-TTCs achieved near-complete tumor regression in trastuzumab-resistant models, and PSMA-TTCs showed marked growth inhibition in mCRPC xenografts. These effects were largely attributed to the ability of alpha-particles to induce irreparable DNA double-strand breaks within a short path length (50–100 µm).

Furthermore, TTCs exhibit enhanced therapeutic potential when combined with agents targeting DNA damage response pathways. Co-administration of TTCs with PARP inhibitors (e.g., olaparib), ataxia telangiectasia and rad3-related protein (ATR) inhibitors, or androgen receptor inhibitors (e.g., darolutamide) have yielded synergistic anti-tumor effects in BRCA-mutant and prostate cancer models. Additionally, alpha-induced immunogenic cell death opens avenues for combining TTCs with immune checkpoint inhibitors, enhancing T-cell infiltration and anti-tumor immunity. Early-phase clinical trials, such as the CD22-targeted TTC (BAY 1862864) in non-Hodgkin lymphoma and PSMA-TTC (BAY 2315497) in metastatic castration-resistant prostate cancer, have demonstrated early signs of efficacy and manageable safety profiles. Overall, TTCs offer a dual mechanism—direct cytotoxicity and immune activation—and are poised to become a cornerstone of targeted alpha therapy in solid and hematologic malignancies.

Hammer et al. [88] demonstrated that a PSMA-targeted thorium-227 conjugate (PSMA TTC, BAY 2315497) shows remarkable preclinical potency against mCRPC models. In vitro, PSMA TTC selectively bound to and was internalized in PSMA-positive cells, inducing double-strand DNA breaks, cell-cycle arrest in G_2_/M, and apoptosis with efficacy correlating to PSMA expression levels. In vivo, single-dose administration (300–500 kBq/kg) led to strong antitumor activity across subcutaneous prostate cancer cell-line and patient-derived xenografts, including models resistant to enzalutamide, with tumor/control (T/C) ratios as low as 0.01–0.31. Notably, in an intratibial bone metastasis model, PSMA TTC inhibited both tumor burden and tumor-induced abnormal bone remodeling demonstrated via bioluminescence, serum PSA reduction, micro-CT, and radiographic analyses, highlighting its potential to target bone-metastatic lesions.

Moreover, PSMA TTC exhibited favorable pharmacokinetics, achieving tumor-specific uptake and retention across various dosing regimens, further validated by robust induction of DNA damage markers (γH2AX) and apoptotic marker cleaved caspase-3 within tumor tissue. In combination studies, the androgen receptor inhibitor darolutamide synergistically enhanced PSMA TTC efficacy: darolutamide upregulated PSMA expression in low-PSMA VCaP xenografts, boosting thorium-227 uptake ~three-fold, increasing DNA damage signaling, and resulting in significantly improved tumor growth control when compared with monotherapies. Together, these compelling preclinical data support the ongoing phase 1 evaluation of PSMA TTC in metastatic castration-resistant prostate cancer.

Hagemann et al. [89] evaluated a novel CD70-targeted thorium 227 conjugate (CD70 TTC) with robust and specific anti-tumor activity in CD70-positive renal cell carcinoma (RCC) models. In vitro, CD70 TTC retained high affinity (EC50 = 0.24 nM) for CD70 and induced potent, dose-dependent cytotoxicity in human 786-O RCC cells at concentrations as low as 2–20 kBq/mL—compared with minimal effects from isotype control TTCs. In vivo, a single intravenous dose of CD70 TTC (500 kBq/kg) in 786-O xenograft-bearing mice led to remarkable tumor uptake (122 ± 42 %ID/g at day 7 versus ~3 %ID/g for control) and dose-dependent tumor growth inhibition. Impressively, complete tumor suppression occurred at ≥300 kBq/kg, with tumor/control ratios down to 0.02 and increased median survival achieved with only modest, reversible hematologic toxicity and no significant weight loss or renal damage.

Mechanistically, CD70 TTC operates via targeted alpha-emission that induces localized DNA double-strand breaks leading to G_2_/M arrest and tumor cell apoptosis, while maintaining favorable pharmacokinetics and minimal normal tissue retention. Autoradiography of tumor sections revealed stark, star-like alpha particle tracks exclusive to CD70 TTC-treated tumors, confirming selective intratumoral delivery. Despite transient reductions in neutrophils and lymphocytes, blood cell counts fully recovered by day 114 post-treatment, and non-hematologic tissues remained unaffected, supporting the safety profile of this approach. These highly encouraging results underscore the therapeutic promise of CD70 TTC for RCC and justify further development in non-clinical species and clinical evaluation.

Hagemann et al. [90] reported targeted TTCs with a major leap forward in precision alpha-particle therapy, harnessing the high LET and short-range cytotoxicity of alpha particles to inflict irreparable DNA double-strand breaks in tumor cells while sparing healthy tissue. The use of the 3,2 HOPO chelator enables stable conjugation of thorium 227 to a variety of tumor-targeting agents including antibodies and peptides expanding beyond the bone-homing Ra 223 (Xofigo, Bayer Healthcare, Whippany, NJ, USA) to target hematologic cancers (e.g., CD22+, CD33+) as well as solid tumors expressing PSMA, mesothelin, FGFR2, and CD70. Preclinical models have demonstrated potent single-dose efficacy, with multiple TTCs achieving complete tumor regressions, durable responses, and survival benefits even in treatment-refractory or resistant settings.

Beyond direct cytotoxicity, TTCs have shown promising synergy with combination therapies leveraging their DNA damage mechanism. Preclinical data reveal enhanced anti-tumor outcomes when TTCs are combined with DNA-damage response inhibitors such as PARP and ATR inhibitors, effectively exploiting synthetic lethality. Furthermore, evidence of immunogenic cell death via release of danger-associated molecular patterns like calreticulin and HSPs suggests TTCs can stimulate adaptive immune responses and may synergize with immune checkpoint inhibitors. Building on this compelling preclinical foundation, several TTCs such as CD22 TTC, PSMA TTC, and MSLN TTC have entered phase 1 trials to evaluate safety, pharmacokinetics, dosimetry, and preliminary efficacy in patients with hematologic and solid malignancies (Table 9).

### 1.4. Beta Emitters

#### 1.4.1. Yttrium-90

Malone et al. [91] investigated whether ^90^Y microspheres, already used clinically for HCC treatment, could activate nano-photosensitizers to enhance cancer cell death beyond ^90^Y treatment alone. The researchers tested this concept using two human HCC cell lines (SNU-387 and HepG2) with varying differentiation levels along with normal hepatocytes (THLE-2) as controls. The nano-photosensitizer was designed to be activated by the Cerenkov radiation naturally emitted by ^90^Y’s beta particles and consisted of titanium dioxide and titanocene labeled with transferrin (TiO2-Tf-TC).

The team found that combining low-activity ^90^Y with the nano-photosensitizer generated more singlet oxygen and hydroxyl radicals and significantly reduced HCC cell viability compared with ^90^Y alone. The poorly differentiated SNU-387 cells showed a clear dose-dependent response, while well-differentiated HepG2 cells did not. Importantly, normal hepatocytes were unaffected, indicating potential tumor selectivity and reduced toxicity. This strategy may be especially valuable for advanced, poorly differentiated HCC, leveraging ^90^Y’s inherent Cerenkov radiation to activate photosensitizers and enhance tumor killing at lower radiation doses than with standard ^90^Y therapy.

Mantry et al. [92] examined the effectiveness and tolerability of selective internal radiation therapy (SIRT) using yttrium-90 (^90^Y) resin microspheres in 111 patients with unresectable hepatocellular carcinoma (HCC) treated at a single institution between 2004–2013. The treatment demonstrated promising survival outcomes, with a median overall survival of 13.1 months and median liver progression-free survival of 9.8 months. Notably, this survival duration was comparable to or better than systemic therapies like sorafenib (10.7 months) and regorafenib (REG) (10.6 months) available at the time. The procedure was generally well-tolerated, with adverse events occurring in 21.5% of patients in one week and 43.4% at three months post-treatment, primarily consisting of abdominal pain, ascites, and nausea.

The study [92] identified several important prognostic factors that could help clinicians select optimal candidates for ^90^Y resin SIRT. ^90^Y resin microspheres are FDA-approved for liver cancer radioembolization and stand out for delivering high-dose beta-radiation directly to tumors while sparing healthy tissue, a feature now being explored for other solid tumors such as metastatic colorectal [93], pancreatic [94], and neuroendocrine cancers [95]. Patients with early-stage disease of Barcelona clinic liver cancer (BCLC A) had significantly longer survival (27.8 months) compared with advanced-stage patients (BCLC C: 9.2 months). Other factors associated with improved outcomes included absence of bilobar disease (23.5 vs. 9.4 months), lack of portal vein thrombosis (16.2 vs. 8.6 months), and absence of ascites (16.6 vs. 10.3 months). Perhaps most remarkably, six patients who received liver transplantation after SIRT as a bridging therapy achieved a median survival of 69.0 months compared with 12.1 months for non-transplant patients, suggesting the treatment’s potential utility in downstaging tumors for transplant eligibility. Patients on concurrent sorafenib had shorter survival, likely due to selection bias, while those who received ^90^Y resin SIRT showed 13 complete and 13 partial responses at six months. These results highlight the value of Y-90 resin SIRT for selected unresectable HCC patients, especially with less advanced disease or awaiting transplant.

Azeredo-da-Silva et al. [93] developed an updated systematic review and network meta-analysis for the clinical effectiveness and safety of SIRT using ^90^Y resin microspheres in patients with chemotherapy-refractory or chemotherapy-intolerant metastatic colorectal cancer (mCRC). The analysis incorporated data from 15 studies (three with SIRT), comparing SIRT with REG, trifluridine-tipiracil (TFD/TPI), and best supportive care (BSC). Studies with SIRT demonstrated the most favorable OS outcome, with an HR of 0.48 (95% credible interval [CrI]: 0.27–0.87) versus BSC and had the highest-ranking probability for OS (SUCRA = 89.2%). Although SIRT did not show statistically significant differences when compared with REG or TFD/TPI, the trend favored SIRT. Due to limited data, PFS could not be evaluated for SIRT, while TFD/TPI showed the best PFS among available data (HR = 0.44, 95% CrI: 0.31–0.62 vs. BSC)

SIRT exhibited a notably better safety profile compared with REG and TFD/TPI. Grade ≥ 3 adverse events (AEs) were rare with SIRT and limited to manageable radioembolization-induced liver disease (10.3%) and isolated cases of hand-foot syndrome (4.8%), mainly when combined with chemotherapy. In contrast, REG and TFD/TPI had higher incidences of severe AEs: 57% of patients experienced grade ≥ 3 drug-related AEs with REG and 37% with TFD/TPI. Moreover, SIRT’s one-time liver-directed treatment avoids the ongoing toxicity of systemic therapies, and recent studies suggest economic and logistical benefits of same-day mapping and treatment. These findings reaffirm the clinical utility of SIRT as a safe, effective, and well-tolerated third-line or salvage option in patients with liver-dominant mCRC, warranting further comparative trials and broader incorporation into treatment algorithms.

Bozkurt at al. [96] evaluated 24 ^90^Y microsphere treatments (glass and resin types) in 19 patients with unresectable intrahepatic (iCCA) or extrahepatic cholangiocarcinoma (eCCA) to determine factors influencing therapeutic response. Patients were assessed using both anatomical (CT/MRI, RECIST 1.1) and metabolic imaging (FDG PET/CT, PERCIST 1.0), with mean OS of 11.9 ± 2.3 months. No significant correlation was found between OS and microsphere type, treated liver lobe, tumor location (iCCA vs. eCCA), tumor diameter, tumor load, or previous treatment history. However, OS was significantly longer in patients categorized as responders (complete response, progressive disease, or stable disease) when compared with non-responders (progressive disease), with mean OS of 21.4 months vs. 5.8 months, respectively (*p* = 0.005). Both microsphere types were safe and well-tolerated with no serious complications observed. Early metabolic responders (assessed via SULpeak) also showed a greater survival benefit.

Quantitative PET metrics revealed that metabolic tumor volume (MTV) and total lesion glycolysis (TLG) significantly declined after therapy (ΔMTV −45.4% ± 12.1, *p* = 0.028; ΔTLG −616.8 ± 689, *p* = 0.031). While changes in SUVmax were not significant, ΔMTV was positively correlated with OS (*p* = 0.032), underscoring its potential as a prognostic biomarker. Heterogeneous perfusion patterns observed in hepatic artery perfusion scintigraphy (HAPS) were visually linked to differential responses: well-perfused tumor regions responded better, whereas poorly perfused regions were more prone to recurrence. Although quantitative HAPS parameters (VOImax/VOImean) did not significantly predict outcomes, visual patterns aligned with metabolic imaging findings. This is the first study to compare resin and glass microspheres in iCCA and eCCA patients, suggesting both are effective and safe, with treatment planning best guided by a multidisciplinary approach integrating tumor perfusion and metabolic characteristics.

Tsang et al. [97] analyzed 49 patients with metastatic liver-dominant neuroendocrine tumors (NETs) who received ^90^Y radioembolization between 2011–2017. The cohort primarily consisted of patients with well-differentiated tumors (86% grade 1–2) from small bowel (41%) or pancreatic (31%) origins, with 69% having liver-only metastases. Most patients had undergone prior surgical resection (63%) and received segmental Y-90 treatment with a median dose of 2.2 GBq. The treatment demonstrated encouraging efficacy with 86% achieving disease control (53% partial response, 33% stable disease) and only 12% experiencing disease progression.

The study showed that ^90^Y radioembolization was well-tolerated with minimal toxicity—only 2% experienced grade 3–4 biochemical toxicities, and 6% had grade 3 abdominal pain. Median overall survival from Y-90 treatment was 27.2 months, which aligns with previously reported ranges of 22–70 months [98]. Importantly, univariable analysis identified three key prognostic factors associated with longer survival: prior surgical resection of the primary tumor, well-differentiated histology, and low Ki-67 proliferation index. Prior surgical resection emerged as the most significant predictor of improved outcomes. These findings support ^90^Y as an effective liver-directed option for metastatic NETs, especially in surgically resected, well-differentiated cases, while highlighting the need for larger prospective trials to refine patient selection (Table 10).

#### 1.4.2. Lutetium-177

Gibbens-Bandala et al. [99] evaluated [^177^Lu]Lu-Bombesin-PLGA (paclitaxel) to introduce a novel nanomedicine that integrates targeted radiotherapy and controlled chemotherapy for bimodal treatment of breast cancer. This system utilizes poly(lactic-co-glycolic acid) (PLGA) nanoparticles co-loaded with the chemotherapeutic agent paclitaxel and radiolabeled with lutetium-177, conjugated to bombesin peptides for specific targeting of gastrin-releasing peptide receptors (GRPR) overexpressed in breast cancer cells. The nanoparticles, approximately 220 nm in diameter, exhibited sustained drug release and high radiochemical purity (>95%). In vitro assays confirmed high cellular uptake in GRPR-positive breast cancer cell lines (T47D and MDA-MB-231), with a marked increase in cytotoxicity when using the dual-loaded formulation compared to paclitaxel or ^177^Lu-Bombesin alone.

In vivo biodistribution studies in murine breast cancer models demonstrated enhanced tumor accumulation and retention of the nanoconjugate due to both passive (EPR effect) and active (bombesin targeting) mechanisms. Tumor-to-muscle and tumor-to-blood ratios were significantly elevated at 72 h post-injection, indicating effective targeting and minimal off-target retention. Therapeutic efficacy studies revealed that the bimodal nanomedicine produced substantial tumor growth delay and increased survival when compared with monotherapy controls. Furthermore, histopathological analyses showed enhanced apoptosis and reduced tumor vascularization in treated tumors. This dual-action strategy underscores the therapeutic advantage of combining localized radiotherapy and sustained chemotherapy within a single nanosystem, presenting a promising approach for aggressive and receptor-positive breast cancer.

Georgiou et al. [100] investigated the therapeutic potential of beta-emitting ^177^Lu-labeled gold nanoparticles ([^177^Lu]Lu-AuNPs) delivered directly into orthotopic U251 human glioblastoma multiforme (GBM) tumors using convection-enhanced delivery (CED) in NOD-Rag1null IL2rgnull mice. The nanoparticles were functionalized with DOTA chelators for stable ^177^Lu radiolabeling and PEG for improved biocompatibility and dispersion. Convection-enhanced delivery enabled precise, intratumoral distribution, overcoming the blood–brain barrier and maximizing local retention of the therapeutic agent. Biodistribution studies confirmed that most of the injected dose remained confined within the tumor for up to 10 days post-delivery, with negligible leakage to peripheral organs, confirming both safety and tumor-specific localization.

Therapeutic efficacy studies demonstrated that mice treated with [^177^Lu]Lu-AuNPs via CED exhibited significantly prolonged survival when compared with control groups (saline, unlabeled AuNPs, and free [^177^Lu]Lu-DOTA), with a median survival extension of over 50%. Magnetic resonance imaging and histological analysis showed marked tumor volume reduction, reduced cell proliferation (Ki-67 staining), and increased apoptosis (cleaved caspase-3 staining) in the treated group. The study highlights the potential of combining nanotechnology and targeted radiotherapy through CED to enhance the therapeutic index for GBM, a notoriously resistant and infiltrative brain tumor. This localized approach minimizes systemic toxicity and could offer a new platform for treating central nervous system malignancies with poor response to conventional therapies.

Graef et al. [101] evaluated the effectiveness of [^177^Lu]Lu-PSMA radioligand therapy in patients with high-grade glioma (HGG), focusing on intratherapeutic dosimetry to assess whether sufficient therapeutic doses could be delivered to tumors. The researchers treated three patients with a median activity of 6.03 GBq of [^177^Lu]Lu-PSMA over two treatment cycles following European Association of Nuclear Medicine guidelines [102] originally developed for prostate cancer treatment. Notably, only three of 20 HGG patients who underwent [^68^Ga]Ga-PSMA PET/MRI imaging were deemed eligible for treatment based on tracer uptake criteria.

The dosimetry results revealed concerningly low tumor doses, with a median of only 0.56 Gy delivered to the tumor tissue—dramatically lower than the therapeutic expectations and far below the 60 Gy cumulative dose typically used in external beam radiation therapy for glioblastoma. The achieved tumor dose was also substantially lower (1/25th) than reported in a previous encouraging case study by Kunikowska et al. [103]. Other organs received relatively low doses: 0.27 Gy to liver, 2.13 Gy to kidneys, 0.76 Gy to salivary glands, and 0.11 Gy whole-body exposure, indicating acceptable safety profiles but insufficient therapeutic effect.

The authors concluded that despite promising initial case reports, [^177^Lu]Lu-PSMA therapy achieved tumor doses well below therapeutic expectations, making the treatment’s effectiveness highly questionable. They noted that PSMA expressions in HGG occur mainly in endothelial cells rather than tumor cells themselves, which may contribute to insufficient radiation distribution within the tumor mass. The study suggests that this theranostic approach, while potentially safe, does not deliver adequate radiation doses to justify its use as a standard treatment for high-grade gliomas.

Sartor et al. [104] conducted the VISION trial, a Phase 3 international, open-label, randomized study enrolling 831 patients with PSMA PET-positive mCRPC who had progressed after at least one androgen-receptor pathway inhibitor and one or two taxane-based chemotherapies. Participants were randomized 2:1 to receive [^177^Lu]Lu-PSMA-617 (7.4 GBq intravenously every 6 weeks for 4–6 cycles) plus standard care (excluding chemotherapy, immunotherapy, radium-223, and investigational agents) or standard care alone. The co-primary endpoints were imaging-based progression-free survival (rPFS) and overall survival (OS). After a median follow-up of about 20.9 months, [^177^Lu]Lu-PSMA-617 significantly prolonged rPFS, with a median of 8.7 months versus 3.4 months in the control arm (hazard ratio [HR] for progression or death 0.40; 99.2% CI, 0.29–0.57; *p* < 0.001). Overall survival was also significantly improved, with a median of 15.3 months versus 11.3 months (HR for death 0.62; 95% CI, 0.52–0.74; *p* < 0.001). All key secondary endpoints, including objective response rate and time to symptomatic skeletal events, also favored [^177^Lu]Lu-PSMA-617 plus standard care. Addition of radioligand therapy increased grade ≥ 3 adverse events (52.7% vs. 38.0%) but did not affect overall quality of life.

Beyond survival, the trial demonstrated robust biochemical and radiologic activity: among treated patients, ~46% had >50% PSA reductions, and ~33% had >80% PSA declines compared with very low PSA responses in the control group (e.g., ~7% with >50% declines). Median duration of therapy was longer in the [^177^Lu]Lu-PSMA-617 arm (~7.6 months vs. ~2.1 months), reflecting delayed progression. The therapy also reduced early symptomatic skeletal events, with median time to first event extended to 11.5 months vs. 6.8 months in controls (HR 0.50). These outcomes with [^177^Lu]Lu-PSMA-617—including nearly doubling of rPFS and a ~4-month median OS benefit—established it as an effective targeted radioligand therapy in a heavily pretreated mCRPC population and supported its subsequent regulatory approvals and adoption into clinical practice guidelines.

Gosewisch et al. [105] presents a novel and clinically practical method for calculating bone marrow (BM)-absorbed doses during radionuclide therapies. The authors compare a standard reference protocol that requires multiple whole-body planar (WB-P) and quantitative SPECT (QSPECT) scans, with a new hybrid protocol (HP). The HP combines three sequential QSPECT acquisitions with only a single WB-P scan, thereby reducing imaging burden. Using data from 10 patients (five with neuroendocrine tumors receiving [^177^Lu]Lu-Octreotate and five with metastatic castration-resistant prostate cancer receiving [^177^Lu]Lu-PSMA-617), the study evaluated BM dosimetry derived from the hybrid and reference protocols. Results demonstrated that the hybrid protocol, especially when the WB-P image was acquired at 48 and 72 h post-injection, produced total BM absorbed dose estimates that deviated by only 1–3% from the reference method—well within clinically acceptable margins.

Importantly, the study found that BM-absorbed doses had different contributing components: blood, remainder of body (ROB), major organs, and tumors. For [^177^Lu]Lu-octreotate therapy, the blood was the dominant contributor (median 59%), whereas for [^177^Lu]Lu-PSMA-617, the ROB (including tumors) had a higher contribution (median 45%), reflecting disease burden and biodistribution patterns. The authors emphasized that a hybrid imaging protocol can simplify clinical workflows without significant loss in dosimetric accuracy. The maximum deviation in total BM dose was less than 6% when late-time-point planar images (≥48h post-injection) were used, making this protocol viable for routine implementation. This approach improves patient comfort, reduces scanner time, and maintains reliable dosimetry, all essential for safe and effective radionuclide therapy planning.

Chang et al. [106] evaluated a promising approach to improve diagnosis and treatment of epithelial ovarian cancer (EOC) using YKL40-targeted radiopharmaceuticals. In a cohort of 76 patients with EOC, YKL40 levels in ascites were significantly elevated in those with serous histologic subtype, high tumor grade, advanced International Federation of Gynecology and Obstetrics stage, recurrence, chemoresistance, and tumor-related death. For instance, ascite YKL40 levels were higher in advanced-stage patients (13,326.1 ± 228.2 pg/mL) compared with early stage (9255.8 ± 900.6 pg/mL, *p* < 0.001), and in chemoresistant patients (13,605.2 ± 197.5 pg/mL) vs. chemosensitive (11,414.8 ± 510.1 pg/mL, *p* = 0.021). High YKL40 levels correlated with significantly reduced disease-free and overall survival (*p* = 0.001 and *p* = 0.008, respectively), supporting YKL40’s prognostic relevance.

To develop a companion theranostic agent, the authors engineered ^111^In and ^177^Lu-labeled DTPA-YKL40 neutralizing antibodies. Radiolabeling purity exceeded 95% for both isotopes for up to 48 h at 4 °C and 37 °C, confirming their stability. In vivo NanoSPECT/CT imaging showed clear tumor localization in xenografted mice at 24 and 48 h post-injection, particularly with [^177^Lu]Lu-DTPA-YKL40/c41. Biodistribution analysis revealed tumor uptake of 4.1 ± 3.8 %ID/g for [^111^In]In-DTPA-YKL40/c41. Therapeutically, [^177^Lu]Lu-DTPA-YKL40/c41 (7.4–29.6 MBq) significantly reduced tumor volume in ES2 and CA5171 xenografts, with up to ~74.9% reduction when compared with controls (*p* < 0.01). No significant histopathological toxicity was observed at therapeutic doses. These findings suggest that radiolabeled YKL40 antibodies, especially [^177^Lu]Lu-DTPA-YKL40/c41, have high potential as companion theranostic agents in EOC, enabling targeted imaging and therapy.

Acar et al. [107] evaluated the predictive value of molecular imaging metrics from Ga-68 PSMA PET/CT in 19 patients with mCRPC undergoing [^177^Lu]Lu-PSMA imaging and therapy (PSMA I&T). Volumetric parameters such as PSMA tumor volume (PSMA-TV) and total lesion PSMA expression (TL-PSMA) were measured pre- and post-therapy using a semi-automatic software. After therapy, PSMA-TV and TL-PSMA values decreased in 63% and 79% of patients, respectively. Mean PSMA-TV dropped from 157 cm^3^ to 98 cm^3^, and TL-PSMA declined from 1863 to 729 cm^3^ (*p* = 0.016). Similarly, SUVmax values decreased from 33.2 to 18.3 (*p* = 0.003), and PSA levels from 182 ng/mL to 50 ng/mL (*p* = 0.040). Complete and partial responses were seen in 10% and 32% of patients, respectively, based on PERCIST criteria

Importantly, decreases in PSMA-TV and TL-PSMA were significantly associated with longer survival. Patients with reduced TL-PSMA had a median survival of 27 months versus 10 months in those with increased TL-PSMA (*p* < 0.001). For PSMA-TV, survival was 28 vs. 14 months (*p* = 0.001). In contrast, PSA and SUVmax responses were not significantly correlated with survival (*p* = 0.206 and *p* = 0.140, respectively), nor was PERCIST-based response (*p* = 0.232). This indicates that molecular volumetric metrics—especially TL-PSMA—are more reliable predictors of response and survival in Lu-177 PSMA therapy than traditional markers. The study supports integrating these metrics into clinical response assessment protocols for better outcome prediction (Table 11).

#### 1.4.3. Copper-67

Huynh et al. [108] evaluated the therapeutic potential of [^67^Cu]Cu-SAR-BBN, a gastrin-releasing peptide receptor (GRPR)-targeting radiopharmaceutical, in a PC-3 prostate cancer xenograft model. The radiotracer was synthesized with >95% radiochemical purity and demonstrated high serum stability (>95% intact at 96 h in PBS and human serum). In vitro binding studies showed specific and time-dependent uptake in GRPR-positive PC-3 cells, reaching 52.2 ± 1.4% of total radioactivity bound at 6 h, with only 6% attributable to nonspecific binding. Internalization remained under 30%, indicating predominant membrane binding. The compound exhibited favorable biodistribution in vivo, with high initial uptake in the GRPR-rich pancreas (22.3 ± 10.5 %ID/g at 1 h) and efficient clearance from non-target organs such as blood, liver, and kidneys.

Therapeutically, six doses of 24 MBq [^67^Cu]Cu-SAR-BBN (total 144 MBq) administered on days 0, 2, 4, 14, 16, and 18 significantly inhibited tumor growth by 93.3% on day 19 when compared with saline controls. Survival was markedly prolonged in treated mice, with median survival exceeding 54 days versus 34.5 days in controls (*p* = 0.0015). Importantly, no significant weight loss or histopathological abnormalities were observed in vital organs (kidney, liver, pancreas), and immunohistochemistry revealed more necrotic tumor regions in treated mice, although Ki-67 proliferation indices were not significantly different. These findings underscore the potential of [^67^Cu]Cu-SAR-BBN as a safe and effective theranostic agent for GRPR-expressing cancers, with copper-67 offering a favorable alternative to traditional radionuclides due to its imaging compatibility with ^64^Cu and suitable half-life for peptide pharmacokinetics.

Hao et al. [109] demonstrated the feasibility of using [^67^Cu]Cu-NOTA-pertuzumab as a dual-function theranostic agent for HER2-positive breast cancer. The authors successfully achieved high radiolabeling yields (>99%) and high specific activity up to 8.6 GBq/mg using high-purity ^67^Cu. The resulting radioconjugate exhibited excellent in vitro stability (≥97% intact in serum after seven days) and retained strong immunoreactivity (80.6%) toward HER2-positive cells. The specific uptake was ~33× higher in HER2+ HCC1954 cells than HER2− MDA-MB-231 cells (*p* < 0.001), confirming target specificity.

Mice bearing HER2+ xenografts were treated with escalating doses (3.7, 7.4, 14.8 MBq) of [^67^Cu]Cu-NOTA-pertuzumab. Tumor growth was significantly inhibited across all treatment groups, with the low dose halting tumor growth for 25 days and higher doses showing more rapid tumor shrinkage. However, toxicity increased with dose: mice receiving 14.8 MBq survived only 11.7 days on average, while the 7.4 MBq group showed 50% survival on day 18. A comparison between high (1.10 GBq/mg) and low (0.056 GBq/mg) specific activity groups (both receiving 7.4 MBq) showed better early tumor uptake and tumor suppression with the higher specific activity, underlining its therapeutic advantage.

SPECT/CT imaging confirmed excellent tumor visualization at day 2 and day 5 post-injection, with tumor uptake increasing dose-dependently: at day 2, tumors showed 0.47 ± 0.13 MBq/mL (3.7 MBq group), 1.31 ± 0.25 MBq/mL (7.4 MBq), and 2.20 ± 0.50 MBq/mL (14.8 MBq). These results establish [^67^Cu]Cu-NOTA-pertuzumab as a promising radioimmunotheranostic agent with potent therapeutic and diagnostic potential when using high-specific-activity ^67^Cu.

Cullinane et al. [110] investigated the therapeutic potential of [^67^Cu]Cu-SarTATE in a somatostatin receptor 2 (SSTR2)-expressing tumor model (AR42J). After a single 5 MBq dose, [^67^Cu]Cu-SarTATE inhibited tumor growth by 75%, while the same dose of [^177^Lu]Lu-TATE inhibited growth by 89%. Median survival increased from 12 days in the control group to 21 days in both treatment groups. Tumor uptake of [^64^Cu]Cu-SarTATE measured via PET and ex vivo biodistribution was 61.8 ± 2.4 %IA/g at 4 h post-injection, with kidney and lung uptake at 16.0 ± 0.7 %IA/g and 12.2 ± 0.8 %IA/g, respectively. Tumor-to-background ratios were exceptionally high: 66 ± 7 at 1 h, rising to 76 ± 7 at 4 h (*p* = 0.027), demonstrating excellent imaging potential.

In a second study from the same team [104], a total of 30 MBq of [^67^Cu]Cu-SarTATE or [^177^Lu]Lu-TATE was administered either as a single dose or in two 15 MBq fractions spaced 14 days apart. Fractionated delivery significantly improved survival: 47 days vs. 36 days for [^67^Cu]Cu-SarTATE (*p* = 0.036), and 46 vs. 29 days for [^177^Lu]Lu-TATE (*p* = 0.040). Both agents were well tolerated with no weight loss exceeding 5%. These results underscore that [^67^Cu]Cu-SarTATE matches the efficacy of the clinical standard [^177^Lu]Lu-TATE and supports the use of the ^64^Cu/^67^Cu theranostic pair for SSTR2-targeted PET imaging and peptide receptor radionuclide therapy. Moreover, the shorter half-life of ^67^Cu (2.58 days) offers a higher dose rate than ^177^Lu, with the added benefit of improved prospective dosimetry using ^64^Cu-based PET imaging.

Ketchemen et al. [111] evaluated [^67^Cu]Cu-trastuzumab as a promising theranostic agent for HER2-positive breast cancer (BC). A key finding is its superior in vitro stability, with [^67^Cu]Cu-NOTA-trastuzumab maintaining 97 ± 1.7% stability in human serum after 5 days, significantly outperforming other constructs like [^67^Cu]Cu-3p-C-NETA-trastuzumab (31 ± 6.2%) and [^67^Cu]Cu-DOTA-trastuzumab (28 ± 4%). This high stability, coupled with a low dissociation constant (Kd) of 2.1 ± 0.4 nM and an immunoreactive fraction of 69.3 ± 0.9%, indicates that radiolabeling effectively preserved the binding characteristics of trastuzumab.

Beyond its favorable in vitro properties, [^67^Cu]Cu-NOTA-trastuzumab demonstrated significant therapeutic efficacy in HER2-positive xenograft models. ImmunoSPECT imaging showed high tumor uptake, with 33.9 ± 5.5 %IA/g in JIMT-1 and 33.1 ± 10.6 %IA/g in BT-474 xenografts at 120 h post-injection. Therapeutically, it achieved substantial tumor growth inhibition (TGI), with 78% TGI in BT-474 xenografts after 28 days (compared with 54% for trastuzumab alone) and an impressive 90% TGI in JIMT-1 xenografts after 19 days (versus 23% for trastuzumab alone). A single injection of approximately 16.8 MBq of [^67^Cu]Cu-NOTA-trastuzumab led to complete remission in six of eight BT-474 xenografted mice. and significantly extended mean survival to over 90 days for BT-474 xenografts and 78 days for JIMT-1 xenografts, compared with 77 and 24 days for trastuzumab-treated groups, respectively. In summary, [^67^Cu]Cu-NOTA-trastuzumab shows exceptional stability, strong tumor targeting, and potent therapeutic effects in HER2-positive BC models, supporting its promise as a theranostic candidate for clinical trials (Table 12).

#### 1.4.4. Iodine-131

Noto et al. [112] aimed to determine the maximum tolerated dose (MTD) and evaluate the preliminary efficacy and safety of high-specific-activity ^131^I meta-iodobenzylguanidine (MIBG) in patients with metastatic and/or recurrent pheochromocytoma or paraganglioma (PPGL) in a phase 1, dose-escalating study. Conducted across three centers, the study enrolled 21 eligible patients. The MTD was established at 296 MBq/kg, primarily due to dose-limiting hematologic toxicities (neutropenia and thrombocytopenia) observed in the next higher dose cohort. The median therapeutic dose administered was 21.13 GBq with a range of 12 to 25.8 GBq, corresponding to a median activity of 240 MBq/kg by actual body weight.

The study demonstrated promising efficacy, with 19% of patients achieving a radiographic partial response based on RECIST criteria; notably, all these responders had received doses greater than 18.5 GBq. Overall, 21% of patients with evaluable responses showed a successful overall response (partial or complete response). Biochemical responses were also significant, with 80% of patients showing complete or partial response for serum chromogranin A and 64% for total metanephrines. Survival rates were encouraging, with 85.7% in 1 year and 61.9% at two years of post-treatment. Treatment was generally manageable, with most adverse events mild to moderate, though 76% experienced grade 3–4 events, primarily hematologic, that resolved within a month. These results demonstrate encouraging efficacy and acceptable safety, supporting high-specific-activity [^131^I]I-MIBG as a promising therapy for metastatic or recurrent PPGL.

Hong et al. [113] investigated the biokinetics of ^131^I in patients with differentiated thyroid cancer (DTC), specifically focusing on remnant normal thyroid tissue (ThyR) and metastatic lymph nodes (mLN), and comparing the effects of recombinant human thyrotropin (rhTSH) administration versus thyroid hormone withdrawal (THW) on these kinetics. The study included a total of 57 patients who received ^131^I therapy, with doses ranging from 3.0 to 7.4 GBq. Thyrotropin stimulation was achieved in 23 patients via rhTSH and in 34 patients via THW. Sequential whole-body scans were acquired on days 1, 2, and 4 (or 5) after I-131 administration to assess biokinetics

A total of 126 lesions across the 57 patients were analyzed. The retention rate and effective half-time demonstrated significant differences between lesion types and TSH stimulation methods. Specifically, the retention rate of mLN was significantly lower than that of ThyR (median 2.6% vs. 12.9%, respectively; *p* < 0.001). Similarly, the effective half-time of mLN was significantly shorter than that of ThyR (median 12.5 h vs. 22.4 h, respectively; *p* = 0.003). Regarding TSH stimulation, the retention rate of ThyR was significantly higher in the rhTSH group when compared with the THW group (median 21.8% vs. 10.8%, respectively; *p* < 0.001). The effective half-time of ThyR also followed this trend, being significantly longer in the rhTSH group (median 33.0 h vs. 19.0 h; *p* < 0.001). In contrast, mLN kinetics were similar between rhTSH and THW, indicating that rhTSH provides comparable radiation delivery to metastatic nodes. Among 24 evaluated patients, ablation was successful in 95.8%. These results highlight distinct tissue-specific kinetics and support rhTSH as an effective alternative to THW for ^131^I therapy in DTC.

Zhang et al. [114] evaluated seven observational studies with 125,591 participants and 13,811 pregnancies to investigate the association between postoperative ^131^I therapy and pregnancy outcomes in female patients with DTC. A key finding is that ^131^I therapy generally shows no significant effect on various adverse pregnancy outcomes. Specifically, there was no significant impact on spontaneous abortion (OR = 1.05, *p* = 0.701), induced abortion (OR = 1.06, *p* = 0.859), overall abortion (OR = 1.07, *p* = 0.098), preterm birth (OR = 1.02, *p* = 0.756), stillbirth (OR = 1.58, *p* = 0.364), or congenital malformation (OR = 1.00, *p* = 0.986). These findings are consistent with previous research and suggest that ^131^I therapy does not increase the risk of these complications.

Further analysis revealed that the cumulative radioiodine dose, whether >3.7 GBq or <3.7 GBq, also had no significant effect on occurrence of on abortion (OR = 0.94, *p* = 0.252) or congenital malformation (OR = 1.05, *p* = 0.752). However, a critical detail emerged regarding the timing of pregnancy after therapy. The study found that patients who waited for an interval of more than one year between their last ^131^I therapy and pregnancy had a significantly lower risk of abortion when compared with those with an interval of less than one year (OR = 0.60, *p* = 0.000). This robust finding, supported by sensitivity and publication bias analyses, strongly suggests that pregnancy is not recommended for DTC patients within one year after ^131^I therapy. These results suggest that while ^131^I does not increase most pregnancy risks, delaying conception for at least one year after therapy is strongly advised to reduce miscarriage risk.

He et al. [115] reported the efficacy and safety of I-131-metaiodobenzylguanidine ([^131^I]I-MIBG) therapy in patients with neuroblastoma across 26 clinical studies involving 883 patients. The pooled objective response rate for [^131^I]I-MIBG monotherapy was 39% (95% CI: 32–47%), while stable disease, progressive disease, and minor response rates were 31%, 22%, and 15%, respectively. In contrast, combination therapies (with chemotherapy or radiosensitizers) yielded a lower pooled objective response rate of 28% (95% CI: 14–44%), but showed a higher stable disease rate of 48%, progressive disease rate of 14%, and minor response rate of 11%. Among chemotherapy combinations, the response rate was slightly higher at 35% (95% CI: 20–52%).

In terms of survival, the pooled one-year and five-year survival rates were 64% (95% CI: 51–75%) and 32% (95% CI: 20–46%), respectively. Hematological toxicities were the most prevalent adverse effects. For monotherapy, thrombocytopenia occurred in 53% and neutropenia in 58% of patients. These rates were significantly higher with combination therapy: thrombocytopenia in 79% and neutropenia in 78% of cases. Despite heterogeneity across studies, no significant publication bias was found. These findings support [^131^I]I-MIBG as a valuable therapeutic option, particularly in individualized treatment strategies for relapsed or refractory neuroblastoma.

Mester et al. [116] examined the effects of ^131^I therapy—commonly used in differentiated thyroid cancer—on dental hard tissues using 48 healthy human maxillary incisors. Teeth were exposed to a solution of 0.6 GBq of ^131^I in artificial saliva for various durations (3–192 h), simulating post-therapy salivary radioiodine exposure. The results demonstrated a progressive increase in radioactivity within dental structures, peaking significantly at 36 h (*p* < 0.05), and, surprisingly, continuing to rise even at 192 h despite radioactive decay. Atomic force microscopy revealed that enamel nanostructure alterations began as early as 6 h post-exposure, with HAp crystallite diameters increasing from 40 nm (healthy) to more than 200 nm by 192 h. Roughness values (Ra and Rq) doubled at 36 h, indicating surface demineralization and loss of compactness.

Similarly, dentin exhibited morphological changes, but with delayed onset, starting after 12 h of exposure. Dentin nanostructure alterations included formation of oval-shaped HAp crystallites and submicron boulder-like formations (200–500 nm) by 192 h. Despite dentin’s deeper location and collagen-rich composition, which initially offered resistance, statistically significant increases in surface roughness at 12 and 48 h (*p* < 0.05) were identified. These findings confirm that ^131^I exposure leads to the breakdown of the crystalline network in both enamel and dentin resulting in structural degradation at micro- and nanoscales. This study provides critical insights into the potential oral health complications associated with radioiodine therapy (Table 13).

## 2. Comparative Analysis: Alpha Versus Beta Emitters

### 2.1. Efficacy

Alpha and beta emitters differ significantly in their LET and path length; these differences influence their therapeutic efficacy. Alpha particles, characterized by high LET (50–230 keV/μm), deliver potent cytotoxic effects over a very short range (50–100 μm), making them particularly effective for eliminating small clusters of cancer cells or micrometastases. Their ability to cause irreparable double-strand DNA breaks results in high tumoricidal potency, even with a few decays per cell [3,16]. In addition, alpha emitter efficacy is less dependent on oxygen levels and cell cycle status; therefore, they can be effective against tumors that are resistant to radiation or chemotherapy. In contrast, beta emitters possess lower LET (0.2–2 keV/μm) and a longer path length (up to 12 mm), allowing them to target larger or heterogeneous tumor masses through the “crossfire effect,” where nearby non-targeted cells receive therapeutic doses due to beta particle spillover [1,3,17].

### 2.2. Safety Profiles

The potent and localized damage caused by alpha emitters limits collateral damage to surrounding healthy tissues, which is advantageous in cases requiring high precision (e.g., hematologic malignancies or minimal residual disease) [10,21,28]. However, their high potency also raises concerns about toxicity if not properly targeted, especially in organs with low repair capacity (e.g., bone marrow or kidneys) [21]. Beta emitters, while generally safer in terms of radiobiological effects per decay, may cause more diffuse tissue damage due to their extended range, especially in non-target tissues, leading to side effects like myelosuppression and organ toxicity in cases of non-specific accumulation [27].

An additional consideration is the pharmacokinetic match between the compound and the radionuclide: for example, the antibody circulation time should be sufficiently long to align with the radionuclide half-life to achieve optimal therapeutic effect [117]. However, prolonged circulation can increase radiation exposure to normal organs and tissues, raising the risk of systemic toxicity [118,119]. Thus, balancing maximum tumor irradiation with minimal damage to healthy tissue through careful selection of radionuclide, antibody engineering, and dosing schedule is a critical factor in treatment design [120].

## 3. Targeting and Off-Target Effects

Successful application of both emitter types depends on precise targeting mechanisms such as monoclonal antibodies, peptides, or small molecules. Alpha emitters, due to their short range, require highly specific and rapid internalization into the target cells to maximize efficacy and minimize toxicity [25,29]. Their low tolerance for off-target localization necessitates robust targeting vectors and rapid clearance from non-target tissues [21,120]. Beta emitters have more flexible suboptimal targeting due to their longer range, allowing for crossfire even when uniform uptake is not achieved. However, this also increases the risk of damaging nearby normal tissues and limits their use in treating microscopic disease [19,27].

### Clinical vs. Preclinical Status

Several beta-emitting radiopharmaceuticals such as [^177^Lu]Lu-DOTATATE [121,122] and [^90^Y]Y-ibritumomab tiuxetan [123,124] are already FDA-approved and widely used in clinical oncology. Beta therapy is well established in neuroendocrine tumors and lymphoma, with ongoing expansion to other indications. In contrast, alpha emitters like [^223^Ra]RaCl_2_ (approved for metastatic prostate cancer) [125,126] represent a more recent clinical innovation. Despite their promise, many alpha emitters (e.g., ^225^Ac, ^211^At, ^213^Bi) remain in the preclinical or early clinical trial stages due to challenges in production, radiolabeling, and dosimetry [21,25]. Nonetheless, their superior cytotoxic profile and potential for overcoming resistance to beta therapy are driving increasing interest in translational and clinical research [29]. Table 14 summaries representative active clinical trials with alpha and beta emitters (the data are adapted from https://clinicaltrials.gov/, accessed on 7 August 2025).

Alpha and beta emitters hold significant promise for the future, especially in the fields of nuclear medicine and radionuclide therapy. Medically, these emitters are at the forefront of targeted radiotherapy, offering new hope for cancer treatment [127]. Alpha emitters such as Actinium-225 and Radium-223 are gaining attention due to their high LET, which allows them to effectively kill cancer cells while minimizing damage to surrounding healthy tissues. This makes them ideal for treating small clusters of cancer cells or micrometastases [17,21]. Beta emitters, including Lutetium-177 and Iodine-131, are already widely used for treating thyroid cancer, neuroendocrine tumors, and prostate cancer. They offer a longer tissue penetration range than alpha particles, making them suitable for larger or more diffuse tumors [128].

Looking ahead, the integration of alpha and beta emitters with advanced targeting molecules such as monoclonal antibodies and peptides promises to make therapies more precise and effective [21,129]. Personalized medicine is also expected to thrive, with radiopharmaceuticals being tailored to the specific biological markers of individual patients’ tumors [17,130]. Outside of medicine, these isotopes are being investigated for use in nuclear batteries and environmental tracing. As production technologies improve and safety protocols advance, alpha and beta emitters will likely play an even more prominent role in both health care and scientific innovation [131,132].

## 4. Challenges

While alpha and beta emitters offer significant potential in medicine and industry, several challenges hinder their broader use. One major issue is the safe handling and containment of these radioactive substances, as their emissions can pose serious health risks if not properly controlled [133]. Alpha emitters, while highly potent in targeting cancer cells, have limited penetration depth requiring precise delivery to tumor sites, which can be technically demanding [2,29]. Beta emitters, although more penetrating, can cause collateral damage to surrounding healthy tissues if not accurately targeted [19]. Additionally, the production and availability of certain therapeutic isotopes, such as Actinium-225 and Lutetium-177, remain limited and costly, hindering widespread clinical use [134,135]. These isotopes are often difficult to produce in large quantities, requiring specialized nuclear reactors or particle accelerators, which are expensive to operate and not widely available. The limited number of production facilities globally creates supply chain obstructions and makes ready access to these isotopes challenging, particularly in low- and middle-income countries [136,137].

Additionally, the short half-lives of many radioisotopes demand rapid production, distribution, and use, adding logistical complexity and cost [138]. Handling and storage also require strict safety protocols and infrastructure, further increasing operational expenses. Regulatory barriers, complex licensing procedures, and challenges in radioactive waste management contribute to delays and increased costs [139]. These factors collectively limit the scalability and accessibility of alpha and beta emitter-based therapies, despite their proven clinical benefits. Overcoming these challenges will require international collaboration, investment in production technologies, and streamlined regulatory pathways. Furthermore, ongoing research is needed to fully understand the long-term biological effects of these treatments and to develop improved targeting mechanisms that minimize side effects while maximizing therapeutic outcomes [10].

## 5. Limitations and Gaps in Current Research

### 5.1. Resistance Mechanisms

Despite the high cytotoxicity of alpha and beta emitters, resistance to radionuclide therapy is an emerging concern. Tumor cells may develop mechanisms to evade radiation-induced apoptosis, including enhanced DNA repair pathways, hypoxia-induced radioresistance, and adaptive antioxidant responses [140]. For beta therapy, the lower LET allows some cancer cells to survive sublethal damage and activate repair mechanisms, leading to treatment failure in heterogeneous or radioresistant tumors [141]. Additionally, downregulation or mutation of molecular targets (e.g., PSMA, somatostatin receptors) can result in decreased uptake of radiolabeled agents, further reducing therapeutic efficacy [142]. These resistance mechanisms are still poorly understood in the context of radiopharmaceutical therapy and require dedicated investigation, particularly in long-term and repeat-dosing scenarios.

### 5.2. Lack of Long-Term Data

One of the most significant gaps in current research is the absence of robust long-term follow-up data, particularly for alpha emitters. Most clinical trials focus on short-term safety and efficacy endpoints with limited information on late toxicities, secondary malignancies, or long-term survival benefits [21]. This is especially critical due to the high LET of alpha particles, which, while potent, may induce off-target effects or long-term damage to surrounding normal tissues [29]. Additionally, data on repeated dosing, cumulative organ toxicity (especially in kidneys, salivary glands, and bone marrow), and quality-of-life outcomes remain sparse. The lack of standardized dosimetry further complicates the ability to assess long-term safety and optimize therapeutic windows [143].

### 5.3. Limitations in Personalized Medicine

While the concept of personalized radiopharmaceutical therapy is gaining traction, its implementation remains limited. The variability in tumor receptor expression, heterogeneity in biodistribution, and differences in clearance rates between patients make it challenging to standardize dosing or predict treatment outcomes [144]. Current protocols often rely on fixed dosing schemes rather than patient-specific dosimetry, particularly in vulnerable populations such as children and pregnant women, potentially leading to suboptimal efficacy or increased toxicity [145]. Furthermore, there is a lack of validated biomarkers to guide radionuclide selection, monitor response, or predict resistance [146]. Personalized treatment strategies require integration of molecular imaging, genomics, and adaptive radiopharmaceutical design, an area that remains underdeveloped due to technological, regulatory, and logistical barriers [130].

### 5.4. Neutron Capture Therapy (NCT) as an Alternative to Alpha and Beta Radionuclide Therapy

Neutron capture therapy (NCT) provides a distinct yet complementary paradigm to established alpha- and beta-particle–based radiotherapies. While alpha and beta therapies rely on the systemic delivery and decay of radionuclides, NCT separates biological targeting from radiation generation [147]. Cytotoxicity is achieved only after neutron irradiation of tumor-localized, non-radioactive capture agents, enabling spatially confined severe linear energy transfer (LET) damage at the cellular or subcellular level [148].

Boron neutron capture therapy (BNCT) closely targeted alpha therapy from a radiobiological standpoint. The ^10^B(n, α)^7^Li reaction produces densely ionizing particles with path lengths comparable to a single cell diameter, resulting in highly localized DNA damage and minimal crossfire effects [149]. Like alpha emitters, BNCT demonstrates high relative biological effectiveness (RBE) and reduced dependence on oxygenation status or cell cycle phase, making it particularly attractive for hypoxic or radioresistant tumors [150]. However, unlike alpha therapy—where decay occurs wherever the radionuclide distributes—BNCT efficacy depends critically on achieving adequate and selective boron accumulation prior to neutron exposure [151]. Heterogeneous intratumoral boron distribution remains a major limitation, as regions with insufficient boron uptake receive little therapeutic benefit despite neutron irradiation [152]. Therefore, developing more tumor-specific boron delivery agents and accurate, real-time dosimetry are essential to reach optimal patient outcomes.

Gadolinium neutron capture therapy (GdNCT) introduces a different biological emphasis. Following neutron capture by ^157^Gd, the emission of Auger and internal conversion electrons results in extremely short-range energy deposition, often confined to nanometer scales [153]. This places the GdNCT conceptually closer to Auger electron therapy than to conventional beta therapy, with therapeutic effectiveness strongly influenced by intracellular and intranuclear localization. Compared with beta emitters, which rely on millimeter-range crossfire to compensate for heterogeneous uptake, GdNCT requires precise molecular targeting to maximize DNA-proximal damage [153]. The dual functionality of gadolinium as both a neutron capture agent and an MRI contrast agent also highlights its theranostic potential, enabling noninvasive assessment of biodistribution and treatment planning—an advantage not universally available in alpha or beta therapies [154,155].

In contrast, technetium-99–based approaches illustrate the limitations of low-LET emitters in highly localized radiotherapy [156]. Although technetium-99 is predominantly a diagnostic radionuclide, its beta and low-energy electron emissions have been explored in select therapeutic contexts. Compared with the high-LET, cell-confined damage produced by neutron capture reactions, technetium-associated radiation aligns more closely with traditional beta therapy, where therapeutic efficacy depends on cumulative dose delivery and crossfire effects [157].

## 6. Conclusions

Targeted radiopharmaceutical therapy (RPT) with alpha and beta emitters has already transformed the therapeutic landscape for several malignancies, demonstrating meaningful improvements in overall survival, progression-free survival, symptom control, and quality of life in clinical practice. The clinical success of beta-emitting agents such as lutetium-177-based therapies and the growing impact of alpha emitters underscore the capacity of RPT to deliver potent, tumor-selective radiation while limiting off-target toxicity. These advances have established RPT as a validated pillar of precision oncology rather than a purely investigational modality. Despite this progress, important challenges remain. An incomplete understanding of resistance mechanisms has limited long-term safety and efficacy data for emerging agents, and the need for more refined patient-selection strategies continues to shape ongoing research. Practical barriers—including isotope supply, manufacturing scalability, reimbursement models, and regulatory complexity must also be addressed to support broader and more equitable access.

Looking ahead, the integration of advanced imaging, molecular profiling, and individualized dosimetry will further optimize therapeutic index and personalize treatment delivery. Continued investment in infrastructure, translational research, and multidisciplinary collaboration will be critical to advancing next-generation radiopharmaceuticals. By building on its demonstrated clinical impact while systematically addressing current limitations, RPT is poised to further improve outcomes for patients with otherwise limited treatment options.

## Figures and Tables

**Table 1:** Alpha vs. beta emitters in radiopharmaceuticals.

Property	Alpha Emitters (α)	Beta Emitters (β)	Refs.
Common radionuclides *	^223^Ra, ^211^At, ^212^Bi, ^225^Ac, ^213^Bi, ^212^Pb, ^149^Tb, ^227^Th	^90^Y, ^131^I, ^177^Lu, ^67^Cu	
Physical half-life	^223^Ra: 11.4 d ^211^At: 7 h ^212^Bi: 60.6 min ^225^Ac: 10 d ^213^Bi: 45.6 min ^212^Pb: 10 h ^149^Tb: 4 h ^227^Th: 18 d	^90^Y: 64 h ^131^I: 8 d ^177^Lu: 6 d ^67^Cu: 61 h	
Type of radiation	Helium nucleus (2 protons + 2 neutrons)	Electron or positron	[17]
Mass	Heavy (~4 amu)	Light (~0.0005 amu)	[17]
Charge	+2	−1 or +1	
LET	High (~50–230 keV/µm)	Low (~0.2 keV/µm)	[3]
RBE	~5 (5–10× higher than beta)	~1	[1,3,17]
Penetration range in tissue	Very short (50–100 µm)	Long (2–12 mm)	[3,18]
Mechanism of action	Dense ionization → double-strand breaks, highly cytotoxic	Sparse ionization → single-strand breaks through free radicals	[3,19]
Tumor targeting requirements	Highly specific targeting required due to high cytotoxicity	More forgiving due to broader energy distribution	[20,21]
Bystander effect	Minimal	Significant due to crossfire effect	[22,23]
Target suitability	Micrometastases, single cells	Larger tumors, heterogeneous cell populations	[20,24]
Advantages	▪ Potent cytotoxicity ▪ Effective against micro metastases ▪ Minimal off-target damage due to short range	▪ Suitable for larger tumors ▪ Easier production ▪ Established clinical use	[20,25,26]
Limitations	▪ Complex production ▪ Limited availability ▪ Radiolysis and short shelf life	▪ Lower cell kill efficiency per decay ▪ Higher collateral damage risk	[16,27]
Regulatory and safety concerns	Potential safety concerns due to toxicity and radiation handling	Well-established regulatory pathway	[28,29,30]
Current clinical applications	▪ ^223^Ra for bone metastases ▪ ^225^Ac-PSMA for prostate cancer	▪ ^131^I for thyroid cancer ▪ ^177^Lu-DOTATATE for neuroendocrine tumors (NETs) ▪ ^90^Y-ibritumomab for lymphoma	[31,32,33,34,35,36,37,38,39,40,41]
Clinical challenges	▪ Chelator development for stable complexation ▪ Dosimetry ▪ Logistics of short-lived isotopes	▪ Limited therapeutic efficacy in micrometastatic or minimal residual disease ▪ Off-target toxicity ▪ Need for personalized dosimetry	[18,21,26,42,43,44,45]
Radiation shielding	Paper, gloves sufficient	Plastic, glass shielding required	[46]

LET = linear energy transfer, RBE = relative biological effectiveness, Ref. = reference number. * ^223^Ra (Radium-223), ^211^At (Astatine-211), ^212^Bi (Bismuth-212), ^225^Ac (Actinium-225), ^213^Bi (Bismuth-213), ^212^Pb (Lead-212), ^149^Tb (Terbium-149), ^227^Th (Thorium-227), ^90^Y (Yttrium-90), ^131^I (Iodine-131), ^177^Lu (Lutetium-177), ^67^Cu (Copper-67).

**Table 2:** Summary of Radium-223 involved radiotherapy.

Study Type	Model/Population	Radiopharmaceuticals	Key Findings	Ref.
Preclinical	PC3, DU145, 22Rv1 prostate cancer cell lines	Radium-223 dichloride	Ra-223 showed extremely high affinity for HAP (K_d_ = 19.2 × 10^−18^ M); minimal cellular uptake; >100-fold survival reduction on Ra-223-loaded HAP; >55% apoptosis; 50% spheroid volume reduction	[48]
Preclinical	Multiple human cancer cell lines (lung, prostate, ovarian, colorectal)	Radium-223 di-chloride	Strong dose- and time-dependent DNA damage (53BP1 foci >20/nucleus); G2/M arrest (up to 68% in HCT116); clonogenic survival decreased to 11% in sensitive lines	[49]
Clinical	31 patients with bone mCRPC	Radium-223 di-chloride + ethinylestradiol (EE) vs. Radium-223 di-chloride alone	PSA reduction in 67% (combination) vs. 23% (monotherapy), *p* = 0.029; no increase in hematologic or GI toxicity; no OS difference. EE may enhance biochemical response to Ra-223 without compromising safety	[50]
Clinical	36 patients with HR+ bone-dominant metastatic breast cancer	Radium-223 di-chloride	49% Disease control rate at 9 months; median PFS 7.4 months; bone-PFS 16.0 months; ORR 54%; no grade 4–5 toxicities. Ra-223 combined with hormonal therapy is safe	[51]
Clinical	1376 men with mCRPC (US)	Radium-223 di-chloride monotherapy vs. combination/layered therapy	Median OS 22.9 months; OS 30.3 for ≥5 cycles vs. 15.3 months for 1–4 cycles; combination therapy OS 26.6 vs. 20.5 months for monotherapy; HR for death 0.45 (≥5 cycles)	[52]
Clinical	67 patients with mCRPC & bone metastases	Radium-223 di-chloride	Elevated baseline ALP was the only independent predictor of poor OS; BSI ≥ 2 and PSA-DT < 3 months associated with worse outcomes; early use favored	[53]
Clinical	64 men with oligometastatic castration-sensitive PCa (bone)	SABR ± Radium-223 di-chloride	No improvement in composite PFS, MFS, or ADT-free survival; grade 3 AEs: 17% (Ra-223 arm); DDR mutations associated with worse PFS	[54]
Clinical	329 patients with mCRPC receiving [^177^Lu]Lu-PSMA	Prior Radium-223 di-chloride vs. Radium-223 di-chloride-naïve	Comparable PFS (16.0 vs. 11.9 months) and OS (17.9 vs. 14.8 months); no negative impact of prior Ra-223	[55]

**Table 3:** Summary of Actinium-225 involved radiotherapy.

Study Type	Model/Population	Radiopharmaceuticals	Key Findings	Ref.
Preclinical	Modeled human organs (hematologic malignancy setting)	Accelerator-produced ^225^Ac with 0.7% ^227^Ac contamination; anti-CD33 antibodies	^227^Ac and daughters contributed <0.02 mGy/MBq to highest-dose organs; cumulative dose <0.04% of total dose from ^225^Ac; ^227^Th was dominant contributor among contaminants	[56]
Preclinical	HER2+ cell lines and SKOV-3 xenograft mice	[^225^Ac]Ac-DOTA-2Rs15d (HER2-targeted sdAb)	High HER2 affinity (Kd ~3.5 nM); strong cytotoxicity (EC_50_ ~3.9 kBq/mL); tumor uptake ~9.6 %IA/g; median survival extended to 101–143 days; kidney is the critical organ for dosimetry	[57]
Preclinical	Prostate cancer xenografts (22Rv1, DU145) and PDX models	[^225^Ac]Ac-DOTA-YS5 (CD46-targeted antibody)	Sustained tumor uptake (up to 31.8 %ID/g); strong DNA damage (γ-H2AX); survival extended up to 131 days (xenografts) and 198 days (PDX); nephrotoxicity at high doses	[58]
Preclinical	U-87 MG glioblastoma cells; mouse tumor models	[^225^Ac]Ac-Au-TADOTAGA (gold nanoparticle nanobrachytherapy)	High radiolabel stability (~80% retention); strong in vitro cytotoxicity (up to 87% cell kill); intratumoral injection yielded high drug retention (60.7 %IA/g) and localized necrosis	[59]
Preclinical	Mouse model of soft-tissue sarcoma	[^225^Ac]Ac-FAPI-46 (Fibroblast activation protein–targeted Actinium-225 radioligand)	Demonstrated significant tumor growth inhibition and prolonged survival in treated mice. Favorable tumor uptake and therapeutic efficacy support potential translation of FAPI-based targeted alpha therapy into clinical evaluation.	[60]
Clinical	20 heavily pretreated men with mCRPC	Tandem therapy: low-dose [^225^Ac]Ac-PSMA-617 + full-dose [^177^Lu]Lu-PSMA-617	≥50% PSA decline in 65%; median PFS 19 weeks, OS 48 weeks; mild xerostomia only; no ≥grade 2 nephrotoxicity	[61]
Clinical	>300 patients with mCRPC (10 studies)	[^225^Ac]Ac-PSMA targeted alpha therapy	~63% achieved ≥50% PSA decline; median OS 9.5–15.9 months; xerostomia common but mostly mild; grade ≥ 3 hematologic toxicity < 10%	[62]
Clinical	Patients with metastatic paragangliomas (somatostatin receptor–positive disease)	[^225^Ac]Ac-DOTATATE (Actinium-225 labeled DOTATATE targeted alpha therapy)	Demonstrated promising efficacy in heavily pretreated metastatic paraganglioma patients, with objective responses and durable disease control. Acceptable safety profile with manageable hematologic and renal toxicity.	[63]
Clinical	Patients with mCRPC	[^225^Ac]Ac-J591 (PSMA-targeting monoclonal antibody labeled with Actinium-225)	Showed dose-dependent antitumor activity with significant PSA declines and radiographic responses. Hematologic toxicities were most common but manageable, supporting further clinical development.	[64]

**Table 4:** Summary of Bismuth-213 involved radiotherapy.

Study Type	Model/Population	Radiopharmaceutical	Key Findings	Ref.
Preclinical	SCID mouse model of disseminated B-cell NHL	[^213^Bi]Bi-rituximab (anti-CD20)	Complete in vitro cell kill at 740 kBq/mL; single 3700 kBq dose cured 75% of mice; efficacy comparable to [^131^I]I-tositumomab and superior to [^90^Y]Y-rituximab	[65]
Preclinical	Mouse model of microscopic IP ovarian cancer (OVCAR-3)	[^213^Bi]Bi-MX35 (NaPi2b-targeted)	Tumor-free fractions: 0.15 (control), 0.55 (3 MBq), 0.78 (9 MBq); clear dose–response; no significant toxicity	[66]
Preclinical	Peritoneal ovarian cancer xenografts	16F12-MISRII mAb labeled with ^89^Zr (PET), ^177^Lu, or ^213^Bi	Brief IP-RIT with 37 MBq [^213^Bi]Bi-16F12 achieved strong tumor reduction; lower hematologic dose vs. β-therapy; tumor-to-blood dose ratio improved to 6	[67]
Preclinical	Ovarian cancer models	Alpha-RIT (^212^Pb/^212^Bi, ^213^Bi) and Auger-RIT (^125^I) ± cholesterol-modifying drugs	Alpha-RIT killed 67–94% of targeted cells; strong bystander effects mediated by lipid rafts, p38/JNK, ROS; statins and raft disruptors reduced efficacy	[68]
Preclinical	Syngeneic melanoma (Cloudman S91, DBA/2 mice)	[^213^Bi]Bi-h8C3 (melanin-targeted) ± anti-PD-1	Combination therapy delayed tumor growth (~1.5×), extended survival, increased CD8^+^ T-cell infiltration; minimal added toxicity	[69]
Clinical	9 patients with recurrent secondary glioblastoma	[^213^Bi]Bi-DOTA-substance P (local intracavitary/intratumoral)	Well tolerated; minimal systemic exposure; median PFS 5.8 months, OS 16.4 months; two long-term survivors (>39, >51 mo)	[70]

**Table 5:** Summary of Bismuth-212 involved radiotherapy.

Study Type	Model	Radiopharmaceutical	Key Findings	Ref.
Preclinical	Murine breast cancer models (4T1, EO771; orthotopic)	[^212^Bi]Bi-MAA	Radiolabeling efficiency ~50%; strong clonogenic kill at ≥92.5 kBq; intratumoral retention 87–93% at 2–4 h; single 925–3700 kBq dose significantly inhibited tumor growth	[71]
Preclinical	In vitro U87 glioblastoma cells	[^212^Pb]Pb-GSH-Ag_2_TeNPs and daughter ^212^Bi	High radiolabeling efficiency (75%); excellent daughter retention (>96% at 24 h); ~25% nuclear localization; strong clonogenic kill with Auger-emitting surrogate ([^111^In])	[72]

**Table 6:** Summary of Astatin-211 involved radiotherapy.

Study Type	Model	Radiopharmaceutical	Key Findings	Ref.
Preclinical	PANC-1 pancreatic cancer xenografts	[^211^At]At-AuNPs-mPEG	High labeling yield (>90%); tumor uptake 2.25 ± 0.67 %ID/g (3 h); rapid clearance from non-target organs; single 1 MBq dose significantly suppressed tumor growth with minimal toxicity	[73]
Preclinical	C6 glioma (rat) and PANC-1 pancreatic tumors (mouse)	[^211^At]At-AuNP-S-mPEG	Excellent tumor retention (>42 h); size-dependent efficacy; 5–30 nm NPs showed superior tumor diffusion and growth inhibition; no systemic toxicity	[74]
Preclinical	PC3 prostate cancer macro- and microtumor mouse models	[^211^At]At-A11 minibody (anti-PSCA)	~42% macrotumor volume reduction; microtumor TFF up to 95%; transient hematologic toxicity at ≤1.5 MBq; durable safety on long-term follow-up	[75]
Preclinical	U-87 MG glioma xenografts	[^211^At]5 (APBA-modified RGD peptide)	Enhanced blood circulation and tumor uptake (10.4 %ID/g at 1 h); dose-dependent tumor suppression; minimal hematotoxicity; high in vivo stability	[76]

**Table 7:** Summary of Lead-212 involved radiotherapy.

Study Type	Model	Radiopharmaceutical	Key Findings	Ref.
Preclinical	SSTR2/Neuroendocrine tumor model	[^212^Pb]Pb-DOTAMTATE	>20 %ID/g sustained to 24 h; 2.4-fold survival increase (single 370 kBq); ~50% complete responses with 3 × 370 kBq; ~79% tumor-free with added 5-fluorouracil	[77]
Preclinical	CSPG4-expressing TNBC mouse models	[^212^Pb]Pb-225.28	High-affinity binding (K_d_ ~0.5 nM); higher tumor uptake vs. control mAbs. Single IV dose (0.30 MBq) significantly inhibited tumor growth vs. control (*p* < 0.01)	[78]
Preclinical	Subcutaneous Panc039 xenografts and orthotopic PDAC3 tumors	[^212^Pb]Pb-376.96	Tumor uptake 14.0 ± 2.1 %ID/g vs. 6.5 ± 0.9 %ID/g control (*p* < 0.001). Single IV dose (0.36–0.73 MBq) significantly inhibited tumor growth	[79]
Preclinical	Hematologic xenografts (mouse), CD37/MEC-2 & Daudi cells	[^212^Pb]Pb-NNV003	Tumor uptake up to 23 %ID/g (Daudi), 16 %ID/g (MEC-2). 91% survival at 28 weeks in Daudi with 90 kBq; higher doses are effective in MEC-2	[80]
Clinical	HER2/Peritoneal metastases (ovarian), *n* = 18	[^212^Pb]Pb-TCMC-trastuzumab	Biomarker (TAG-72) reduction up to 66% correlated with dose; mild and transient adverse events; all patients progressed ≤8 months	[81]

**Table 8:** Summary of Terbium-149 involved radiotherapy.

Study Type	Model	Radiopharmaceutical	Key Findings	Ref.
Preclinical	PC-3 PIP xenografts (mouse)	[^149^Tb]Tb-PSMA-617	Significant tumor growth delay in all dosing regimens; best efficacy with 3 MBq on days 0 + 1; median survival increases from 20 to 36 days	[82]
Preclinical	AR42J tumor-bearing mice; SSTR/Neuroendocrine tumor model	[^149^Tb]Tb-DOTANOC	PET/CT at 2 h p.i. (~7 MBq) showed high tumor uptake with low renal/background activity	[83]

**Table 9:** Summary of Thorium-227 involved radiotherapy.

Study Type	Model	Radiopharmaceutical	Key Findings	Ref.
Preclinical	Immunocompetent MC38-hMSLN tumors (mouse); in vitro human cancer lines	Th-Mesothelin-TTC (MSLN-TTC)	Monotherapy inhibited tumor growth (T/C ~0.38); strong synergy with anti-PD-L1 (T/C ~0.08); strong rationale for combining MSLN-TTC with checkpoint inhibitors	[84]
Preclinical	MC-38 syngeneic colorectal cancer model	PD-L1-TTCs	Single 500 kBq/kg dose inhibited tumors; TTC plus anti-PD-1 achieved complete, durable tumor eradication; rechallenge resistance	[85]
Preclinical	CDX & PDX solid tumors	MSLN-TTC (BAY 2287411)	Single 500 kBq/kg caused strong regressions (T/C 0.02–0.1); survival benefit; reversible leukopenia only	[86]
Preclinical	Multiple solid & hematologic cancers	TTCs	Significant anti-tumor efficacy, including resistant or metastatic tumors; enhanced therapeutic potential when combined with inhibitors	[87]
Preclinical	mCRPC xenografts, PDXs, bone metastasis models	PSMA-TTC (BAY 2315497)	Single 300–500 kBq/kg achieved T/C 0.01–0.31; strong bone-metastasis control; synergy with darolutamide	[88]
Preclinical	CD70^+^ renal cell carcinoma (786-O)	CD70-TTC	Tumor uptake 122 ± 42 %ID/g; complete suppression at ≥300 kBq/kg; reversible hematologic toxicity	[89]
Preclinical	Hematologic & solid tumors (PSMA, MSLN, FGFR2, CD70, CD22)	TTC platform	Single-dose cures, durable responses in refractory models; multiple TTCs in phase-1 trials	[90]

**Table 10:** Summary of Yttrium-90 involved radiotherapy.

Study Type	Model	Radiopharmaceutical	Key Findings	Ref.
Preclinical	SNU-387, HepG2; normal THLE-2 cell lines	^90^Y + TiO_2_-Tf-TC	Enhanced ROS generation and reduced viability vs. ^90^Y alone; dose-dependent effect in poorly differentiated cells. Demonstrates Cerenkov-activated phototherapy	[91]
Clinical	*n* = 111 patients (2004–2013), Unresectable hepatocellular carcinoma (HCC)	^90^Y resin microsphere SIRT	Median OS: 13.1 months; Median liver PFS: 9.8 months; Comparable or superior to sorafenib (10.7 months) and regorafenib (10.6 months); 13 CR + 13 PR at 6 months, Improved survival with BCLC A	[92]
Clinical	Metastatic colorectal cancer (mCRC), chemo-refractory/intolerant	^90^Y resin SIRT vs. REG, TFD/TPI, BSC	Supports SIRT as safe, effective third-line/salvage therapy for liver-dominant mCRC; logistical and economic advantages	[93]
Clinical	iCCA & eCCA) patients (*n* = 19)	^90^Y resin vs. glass microspheres	Mean OS: 11.9 ± 2.3 months; responders vs. non-responders: 21.4 vs. 5.8 months (*p* = 0.005). Metabolic response (MTV, TLG decline) correlated with OS	[96]
Clinical	*n* = 49 (2011–2017), Patients with metastatic neuroendocrine tumors (NETs)	Segmental ^90^Y radioembolization	Disease control: 86% (53% PR, 33% SD); Median OS: 27.2 months	[97]

**Table 11:** Summary of Lutetium-177 involved radiotherapy.

Study Type	Model	Radiopharmaceutical	Key Findings	Ref.
Preclinical	Breast cancer (GRPR-positive) in vitro and in vivo murine models	[^177^Lu]Lu-Bombesin–PLGA (paclitaxel) nanoparticles	Dual-loaded system showed greater cytotoxicity than paclitaxel or ^177^Lu-Bombesin alone; enhanced tumor uptake and retention; significant tumor growth delay and survival benefit	[99]
Preclinical	Orthotopic U251 GBM mouse model	^177^Lu-labeled gold nanoparticles (^177^Lu-AuNPs)	Median survival increased by >50%; marked tumor volume reduction; increased apoptosis and reduced proliferation	[100]
Clinical	High-grade glioma (HGG); *n* = 3 treated patients	[^177^Lu]Lu-PSMA	Median tumor dose 0.56 Gy; ~25× lower than prior case report, suggesting insufficient therapeutic efficacy of PSMA RLT in HGG	[101]
Clinical	Patients with mCRPC previously treated with androgen receptor pathway inhibitors and taxane chemotherapy	[^177^Lu]Lu-PSMA-617 (Lutetium-177–labeled PSMA-targeted radioligand therapy)	[^177^Lu]Lu-PSMA-617 significantly improved overall survival and radiographic progression-free survival compared to standard care alone. Higher PSA response rates were observed. Adverse events were more frequent in the treatment group but were generally manageable, supporting its role as an effective therapy in advanced mCRPC.	[104]
Clinical	*n* = 10 patients (NET & mCRPC)	[^177^Lu]Lu-Octreotate, [^177^Lu]Lu-PSMA-617	BM dose estimates deviated only 1–3% from reference protocol; max deviation < 6%. Enables clinically practical, accurate BM dosimetry for routine Lu-177 therapies	[105]
Preclinical/Clinical	EO patients (*n* = 76) + ES2 and CA5171 xenograft models	[^111^In]/[^177^Lu]Lu-DTPA-YKL40/c41, [^111^In]In-DTPA-YKL40/c41	High YKL40 correlated with poor prognosis; [^177^Lu]Lu-DTPA-YKL40 reduced tumor volume by ~75% in vivo	[106]
Clinical	mCRPC patients (*n* = 19)	[^177^Lu]Lu-PSMA with Ga-68 PSMA PET/CT	Decreases in PSMA-TV and TL-PSMA strongly correlated with OS (27–28 vs. 10–14 months); PSA/SUVmax not predictive	[107]

**Table 12:** Summary of Copper-67 involved radiotherapy.

Study Type	Model	Radiopharmaceutical	Key Findings	Ref.
Preclinical	PC-3 prostate xenograft model	[^67^Cu]Cu-SAR-BBN	Six doses (24 MBq each) inhibited tumor growth by 93.3%; median survival > 54 days vs. 34.5 days in controls; immunohistochemistry showed increased necrosis	[108]
Preclinical	Mice bearing HER2+ xenografts	[^67^Cu]Cu-NOTA-pertuzumab	Tumor growth halted or regressed dose-dependently; higher specific activity improved tumor uptake and suppression; SPECT/CT showed clear tumor visualization	[109]
Preclinical	SSTR2 tumor models (AR42J)	[^67^Cu]Cu-SarTATE	Single 5 MBq dose inhibited tumor by 75%; fractionated 30 MBq dose increased survival to 47 days; PET imaging tumor uptake 61.8 ± 2.4 %IA/g at 4 h; tumor-to-background ratios > 70. Comparable efficacy to clinical [^177^Lu]Lu-TATE	[110]
Preclinical	HER2-positive xenograft models	[^67^Cu]Cu-NOTA-trastuzumab	Single ~16.8 MBq injection led to 78–90% tumor growth inhibition, complete remission in 6/8 BT-474 mice; median survival > 90 days (BT-474), 78 days (JIMT-1). Excellent tumor targeting and potent therapeutic effect	[111]

**Table 13:** Summary of Iodine-131 involved radiotherapy.

Study Type	Model	Radiopharmaceutical	Key Findings	Ref.
Clinical	Metastatic/recurrent pheochromocytoma & paraganglioma (PPGL), Phase 1, *n* = 21	[^131^I]I-MIBG	19% radiographic PR; 21% overall response; 1-year survival 85.7%, 2-year 61.9%; responders received >18.5 GBq; supports use of high-SA ^131^I-MIBG in PPGL therapy	[112]
Clinical	DTC patients, *n* = 57	^131^I	Metastatic nodes (mLN) had lower retention (2.6% vs. 12.9%) and shorter half-time than remnant thyroid tissue (ThyR); rhTSH increased ThyR retention (21.8% vs. 10.8%); successful ablation 95.8%, rhTSH provides an effective alternative to THW	[113]
Clinical	Meta-analysis; 7 studies, *n* = 125,591 participants, 13,811 pregnancies. DTC in women; pregnancy outcomes	^131^I	No significant effect on abortion, preterm birth, stillbirth, congenital malformation; delayed conception >1-year post-therapy reduced abortion risk (OR 0.60), Pregnancy safe after ^131^I if delayed ≥1 year; timing critical for miscarriage risk reduction	[114]
Clinical	Patient with Neuroblastoma, *n* = 883.	^131^I-MIBG monotherapy and combination therapy	Monotherapy ORR 39%; SD 31%, PD 22%, minor response 15%; combo therapy ORR 28%; 1-year survival 64%, 5-year 32%. ^131^I-MIBG is effective salvage therapy; combination regimens increase stable disease, but higher hematologic toxicity	[115]
Clinical	Healthy human maxillary incisors, *n* = 48,	^131^I	Enamel and dentin nanostructure altered; enamel HAp crystallites increased 40 nm to >200 nm; surface roughness doubled; dentin changes delayed but significant	[116]

**Table 14:** Representative clinical trials with alpha and beta emitters.

Clinical Trial Registration No.	Radiotherapeutics	Phase of Study	Type of Emitter	Disease Indications
NCT04506567	[^225^Ac]Ac-J591	I/II	α	Progressive mCRPC
NCT04597411	[^225^Ac]Ac-PSMA-617	I	α	PSMA-positive prostate cancer
NCT04946370	^225^Ac-J591	I/II	α	Prostate cancer
NCT05595460	[^225^Ac]DOTATATE (RYZ101)	I	α	Small cell lung cancer
NCT05983198	[^225^Ac]Ac-PSMA-R2	I/II	α	mHSPC, mCRPC
NCT06147037	[^225^Ac]-FPI-2068	I	α	Advanced solid tumors
NCT06590857	[^225^Ac]DOTATATE (RYZ101)	I/II	α	Breast cancer
NCT06726161	[^225^Ac]RYZ801	I	α	Hepatocellular carcinoma
NCT06975332	[^225^Ac]Ac-DOTA-SP	Not Applicable	α	Glioma
NCT07150806	[^225^Ac]DOTATATE (RYZ101)	I/II	α	Recurrent meningioma
NCT04083183	[^211^At]At-BC8-B10	I/II	α	Nonmalignant diseases
NCT06441994	[^211^At]At-PSMA-5	I	α	Prostate cancer
NCT04997317	^177^Lu-DOTA-JR11, ^177^Lu-DOTATOC	I/II	β	Meningiomas
NCT05204927	[^177^Lu]Lu-PSMA-I&T	III	β	Metastatic castration-resistant prostate cancer
NCT05249114	Lu-177 DOTATATE	I	β	Neuroendocrine tumors
NCT05477576	[^177^Lu]SSA	III	α	Neuroendocrine tumors
NCT05691465	[^177^Lu]Lu-DOTATATE	II	β	Metastatic prostate cancer
NCT06084338	Lutetium Lu 177 vipivotide tetraxetan	II	β	mCRPC
NCT06197139	[^177^Lu]Lu-XT117	I	β	Advanced solid tumor
NCT06211647	[^177^Lu]Lu-XT117	I	β	Advanced solid tumor
NCT05153772	[^212^Pb]Pb-DOTAMTATE	II	α	Neuroendocrine tumors

mCRPC = metastatic castration-resistant prostate cancer. mHSPC = metastatic hormone-sensitive prostate cancer.

## Data Availability

The materials and resources in this study are available from the corresponding author upon request.
